# B-to-A transition in target DNA during retroviral integration

**DOI:** 10.1093/nar/gkac644

**Published:** 2022-08-10

**Authors:** Ilona K Jóźwik, Wen Li, Da-Wei Zhang, Doris Wong, Julia Grawenhoff, Allison Ballandras-Colas, Sriram Aiyer, Peter Cherepanov, Alan N Engelman, Dmitry Lyumkis

**Affiliations:** The Salk Institute for Biological Studies, La Jolla, CA 92037, USA; Department of Cancer Immunology and Virology, Dana-Farber Cancer Center, Boston, MA 02215, USA; Department of Medicine, Harvard Medical School, Boston, MA 02115, USA; Department of Cancer Immunology and Virology, Dana-Farber Cancer Center, Boston, MA 02215, USA; Department of Medicine, Harvard Medical School, Boston, MA 02115, USA; Institute of Bioinformatics and Medical Engineering, School of Electrical and Information Engineering, Jiangsu University of Technology, Changzhou 213001, China; Department of Cancer Immunology and Virology, Dana-Farber Cancer Center, Boston, MA 02215, USA; Department of Cancer Immunology and Virology, Dana-Farber Cancer Center, Boston, MA 02215, USA; Chromatin Structure and Mobile DNA Laboratory, The Francis Crick Institute, London NW1 1AT, UK; The Salk Institute for Biological Studies, La Jolla, CA 92037, USA; Chromatin Structure and Mobile DNA Laboratory, The Francis Crick Institute, London NW1 1AT, UK; Department of Infectious Disease, St-Mary's Campus, Imperial College London, London W2 1PG, UK; Department of Cancer Immunology and Virology, Dana-Farber Cancer Center, Boston, MA 02215, USA; Department of Medicine, Harvard Medical School, Boston, MA 02115, USA; The Salk Institute for Biological Studies, La Jolla, CA 92037, USA; Department of Integrative Structural and Computational Biology, The Scripps Research Institute 10550 N Torrey Pines Rd, La Jolla, CA 92037, USA; Graduate School of Biological Sciences, Section of Molecular Biology, University of California San Diego, La Jolla, CA 92093, USA

## Abstract

Integration into host target DNA (tDNA), a hallmark of retroviral replication, is mediated by the intasome, a multimer of integrase (IN) assembled on viral DNA (vDNA) ends. To ascertain aspects of tDNA recognition during integration, we have solved the 3.5 Å resolution cryo-EM structure of the mouse mammary tumor virus (MMTV) strand transfer complex (STC) intasome. The tDNA adopts an A-like conformation in the region encompassing the sites of vDNA joining, which exposes the sugar-phosphate backbone for IN-mediated strand transfer. Examination of existing retroviral STC structures revealed conservation of A-form tDNA in the analogous regions of these complexes. Furthermore, analyses of sequence preferences in genomic integration sites selectively targeted by six different retroviruses highlighted consistent propensity for A-philic sequences at the sites of vDNA joining. Our structure additionally revealed several novel MMTV IN-DNA interactions, as well as contacts seen in prior STC structures, including conserved Pro125 and Tyr149 residues interacting with tDNA. In infected cells, Pro125 substitutions impacted the global pattern of MMTV integration without significantly altering local base sequence preferences at vDNA insertion sites. Collectively, these data advance our understanding of retroviral intasome structure and function, as well as factors that influence patterns of vDNA integration in genomic DNA.

## INTRODUCTION

Retroviral particles harbor two copies of single-stranded plus-sense genomic RNA, which are converted by reverse transcription into a linear, double-stranded DNA molecule. A defining step in the retroviral replication cycle is the integration of viral DNA (vDNA) into a host cell chromosome. Integration is mediated by the viral integrase (IN) protein, which catalyzes sequential 3′-processing and strand transfer reactions. During 3′-processing, IN site-specifically cleaves the vDNA ends adjacent to invariant CpA dinucleotides, yielding recessed CpA_OH_-3′ termini. During strand transfer, IN uses the vDNA CpA_OH_-3′ termini to cut opposing target DNA (tDNA) strands in a staggered fashion (separated by 4–6 bp, depending on the retroviral species), which covalently links the vDNA 3′ termini to the host genome. Cellular machinery is thought to repair the hemi-integrant (1), resulting in a 4–6 bp target site duplication (TSD) flanking the integrated provirus. Readers are referred to recent comprehensive reviews of retroviral integration (2,3).

Retroviral INs are composed of three canonical structural domains: an α-helical N-terminal domain (NTD), the catalytic core domain (CCD, which adopts an RNase H-like fold and harbors the enzyme active site), and the C-terminal domain (CTD, featuring an SH3-like β-barrel fold) (2,3). The IN active site, which is composed of invariant Asp and Glu residues comprising the DDE motif, is conserved among a wider group of DNA strand-transferases including long terminal repeat (LTR) retrotransposons and some DNA transposases (4). 3′-Processing and strand transfer are one-step transesterification reactions catalyzed by a single IN active site. The active site carboxylates coordinate two divalent metal ions (Mg^2+^ under physiologic conditions), which activate attacking nucleophiles (a water molecule during 3′-processing and a 3′ vDNA hydroxyl group during strand transfer) and destabilize scissile phosphodiester bonds (2,3).

The integration of two vDNA ends requires formation of a multimeric IN complex. In cells, integration is catalyzed by the pre-integration complex (PIC), which is a large nucleoprotein assembly that includes the vDNA and a number of viral and cellular proteins (5,6). PICs, however, are present in very low abundance in cell extracts and therefore are not readily amenable to structural studies. IN proteins produced from recombinant sources can assemble with oligonucleotide mimics of vDNA to form stable and catalytically competent complexes, and pioneering studies with prototype foamy virus (PFV) IN defined the nucleoprotein complexes that support IN 3′-processing and strand transfer activities *in vitro*. Initially, IN binds to and bridges a pair of vDNA ends to form the stable synaptic complex (SSC). Processing of the vDNA ends by IN subsequently yields the cleaved synaptic complex (CSC) (7). When supplied with oligonucleotides that serve as the tDNA, CSCs can form a target capture complex (TCC) (8). Finally, the integration of vDNA ends into bound tDNA converts the TCC into the strand transfer complex (STC) (8). This series of stable nucleoprotein complexes is collectively referred to as intasomes. Harboring covalently joined vDNA and tDNA, the STC intasome can inform the structural bases of IN strand transfer activity and nucleotide sequence selectivity at sites of vDNA insertion.

Retroviruses employ varying numbers of IN molecules to construct their intasomes. Simiispumaviruses, typified by PFV (7–9), as well as δ-retroviruses that include human lymphotropic virus 1 (HTLV-1) (10,11), employ four IN molecules, while α-retroviruses (12,13) and β-retroviruses, the latter of which include mouse mammary tumor virus (MMTV) (14), use eight. The lentiviruses, such as human immunodeficiency virus 1 (HIV-1) and maedi-visna virus (MVV), use 12 or 16 IN molecules (15–18) to form the largest intasomes characterized to date. Prior to this work, intasome STC structures were reported for PFV (8), Rous sarcoma virus (RSV) (12), HIV-1 (17), HTLV-1 (10) and MVV (19). While the PFV and RSV structures were determined by X-ray diffraction, the HIV-1, HTLV-1 and MVV structures were determined by single particle cryogenic electron microscopy (cryo-EM). Despite differences in IN multimerization, all intasomes harbor a conserved intasome core that is constructed from the two catalytic IN subunits as well as a pair of synaptic IN CTDs that help to bridge the catalytic subunits together [reviewed in (20)].

Retroviral integration in host cell genomes is non-random, with preferences observed at the genomic (e.g. transcription units, promoter regions, etc.) as well as local tDNA sequence levels. Host proteins that interact with IN and viral capsid can target PICs to preferred genomic loci, which is best understood for the lentiviruses and the γ-retrovirus Moloney murine leukemia virus (MLV) [see (21) for a recent review]. Interactions between IN and tDNA can moreover influence nucleotide preferences at the sites of vDNA joining (22–28).

Widening of the tDNA major groove accommodates scissile phosphodiester bonds at the PFV IN active sites for strand transfer (8). Substitution of PFV IN CTD residue Arg329, which makes base-specific contacts within the widened major groove, altered local base preferences in *in vitro* integration products (8). Alteration of residue Ala188 within the PFV IN CCD also impacted the base composition of integration sites (8). Ala188 is chemically conserved as a small amino acid residue (Pro, Thr or Ser) across retroviral INs (26). Substitutions of analogous HIV-1 IN Ser119 (25,27), RSV IN Ser124 (29) and MLV IN Pro187 (30) residues could likewise alter local base compositions of integration sites (25,27,30) or yield distinct patterns of target site preferences (29). In some cases, IN Ser119 substitutions reduced the preference of HIV-1 to integrate into gene-dense regions of chromosomes, subtly influencing integration site targeting at the genomic level (25).

We have used MMTV as a model system to investigate retroviral intasome structure and function (14). MMTV has historically provided important insights into mechanisms of virally induced malignant transformation in mouse breast tissues (31) and has received recent attention due to its high degree of similarity to a betaretrovirus implicated in human autoimmune disease and cancer (32,33). Previously, we determined the structure of the MMTV CSC at ∼5–6 Å resolution (14), which revealed that flanking IN protomers can contribute to intasome assembly, but was insufficient to accurately describe structural interfaces, such as IN-vDNA contacts. Moreover, because the CSC did not include the tDNA component, the structure did not explain how MMTV intasomes engage their targets. Thus, our current study had several goals. First, we aimed to improve the resolution of the MMTV intasome in order to comprehensively characterize important IN–IN and IN–vDNA interactions that were missed in the initial lower-resolution structure. Second, we wanted to investigate how the intasome engages tDNA to better define the rules underlying tDNA binding and IN strand transfer functionality. We have accordingly determined the structure of the MMTV STC resolved to 3.5 Å resolution, with more acute 3 Å resolution in the intasome core region. The structure revealed a pronounced bend in tDNA, which transitions from B-form DNA in peripheral regions to the A-form in and around the sites of vDNA joining. Comparative analysis with existing STC structures revealed that both tDNA bending and A-like character appear to be general features of retroviral IN-engaged tDNA. To extend these findings to intasome function in cells, we examined sequence preferences at integration sites from six different retroviruses, which highlighted the consistent propensity to select for sequences with enhanced A-form characteristics at the sites of vDNA joining. Enzymatic assays and viral infectivity experiments using IN mutants showed that alterations of IN CCD residue Pro125 did not significantly alter local base preferences at sites of MMTV DNA joining and that flanking IN dimers play a critical role in the concerted integration of two vDNA ends. The collective data enhances our understanding of MMTV IN structure/function and highlights the general requirement for A-form tDNA during retroviral integration.

## MATERIALS AND METHODS

### Plasmid DNAs

MMTV IN with an N-terminal hexahistidine (His_6_) tag was previously expressed in *Escherichia coli* strain PC2 from plasmid pCPH6P-MMTV-IN, which yielded approximately 1 mg of purified protein per l of induced cell culture (14,34). In an attempt to improve protein yield, MMTV IN was expressed from a pET-SUMO (Invitrogen) or pMAL-c5x (New England Biolabs) plasmid backbone as a His_6_-SUMO or maltose-binding protein (MBP) N-terminal tag fusion protein, respectively. Because the MBP tag improved MMTV IN yield by approximately 2-to-3-fold, all proteins in the present study were expressed as fusions to MBP. The cleavage site for factor Xa protease encoded in pMAL-c5x vectors was changed by PCR-directed mutagenesis to the site recognized by human rhinovirus (HRV) 3C protease, yielding plasmid pMAL-c5x-HRV3C-MMTV IN. IN mutant expression plasmids were created by mutating pMAL-c5x-HRV3C-MMTV IN using PCR-directed mutagenesis. Cleavage with HRV3C protease predictably yielded IN N-terminal GPALES sequences, with the heterologous GP dipeptide derived from the protease recognition site.

MMTV for infection assays was produced from cells by transfection with a 4-plasmid system essentially as previously described (35). The enhanced green fluorescent protein sequence encoded within MMTV transfer vector pRRpCeGFPWPRE25 (35) was swapped for the firefly luciferase gene, yielding pRRpCLucWPRE25. The MMTV packaging plasmid pCMgpRRE17, which encodes the virion structural proteins and replication enzymes including IN, was as described (35). Viral mutant IN expression constructs were created by mutating pCMgpRRE17 using PCR-directed mutagenesis. The coding sequences of all plasmids that were synthesized by PCR were examined by dideoxy sequencing to verify the presence of site-directed mutations and the absence of unwanted secondary changes. Expression plasmids for HIV-1 Rev (pRSV-Rev) and vesicular stomatitis virus glycoprotein G (VSV-G; pCG-VSV-G) were previously described (36,37).

### MMTV IN expression and purification

A colony of PC2 bacteria transformed with pMAL-c5x-HRV3C-MMTV IN DNA was inoculated into 100 ml NB media (10 g tryptone, 5 g yeast extract, 5 g NaCl, 2 g glucose, 0.1 g ampicillin/l), and the culture was grown overnight at 250 rpm at 37°C. Cells were diluted at 1:60 the following day into 6 l of fresh NB media, and 8 flasks containing 750 ml each were grown at 30°C, 250 rpm until reaching an optical density at 600 nm of 0.6, at which time isopropyl β-d-1-thiogalactopyranoside and ZnCl_2_ were added to the final concentrations of 0.4 mM and 50 μM, respectively. Following an additional 4 h of growth at 30°C and 250 rpm, the bacteria were harvested by centrifugation, and the pellets were stored at -80°C.

Thawed bacterial pellets resuspended in 25 ml MMTV IN extraction buffer (EB; 20 mM HEPES, pH 7.6, 1 M NaCl, 5 mM 3-((3-cholamidopropyl) dimethylammonio)-1-propanesulfonate (CHAPS), complete EDTA-free protease inhibitor (Millipore Sigma)) were sonicated on ice for 6 min at 20 mA using repetitive cycles of 5 s on and 10 s off. Amylose affinity chromatography was performed by gravity flow using Econo-Pac chromatography columns (Bio-Rad). The bacterial lysate, clarified by centrifugation at 15 000 g for 1 h at 4°C, was added to 6 ml of amylose resin (New England Biolabs) that had been pre-equilibrated with 30 ml of EB. After washing the columns with 60 ml EB, beads resuspended in 10 ml EB were incubated with 880 μg HRV3C protease for 2 days at 4°C with mild 20 rpm agitation. EB (60 ml) was used to elute proteins from post-cleavage beads. Protein eluates concentrated ∼6-fold by ultrafiltration using 10 kDa molecular weight cutoff units were diluted 1:5 in heparin column buffer (HCB; 20 mM HEPES pH, 7.6, 5 mM CHAPS, 2 mM dithiothreitol (DTT)). The diluted sample was loaded onto a 5 ml HiTrap Heparin column (Cytiva) that was pre-equilibrated with HCB containing 0.1 M NaCl. After washing the column with 25 ml of HCB–0.1 M NaCl, proteins were eluted using a linear gradient of 0.1 M to 1.5 M NaCl in HCB. Column fractions containing MMTV IN were pooled and concentrated by ultrafiltration to ∼300 μl, which was then applied to a Superdex 200 10/300 GL gel filtration column (Cytiva) equilibrated with 20 mM HEPES, pH 7.6, 1 M NaCl, 5 mM CHAPS, 2 mM DTT, 0.5 mM EDTA. Following gel filtration chromatography, IN-containing fractions were pooled, concentrated by ultrafiltration to ∼10 mg/ml, dialyzed against buffer containing 20 mM HEPES, pH 7.6, 1 M NaCl, 5 mM CHAPS, 2 mM DTT, 0.5 mM EDTA, 10% glycerol, flash frozen in liquid N_2_, and stored at −80°C. Purification and final concentration profiles of mutant INs did not noticeably differ from wild type (WT) MMTV IN, indicating the analyzed amino acid substitutions did not grossly affect IN tertiary structure.

### MMTV intasome assembly

The branched DNA (bDNA) substrate was formed by annealing the following three DNA strands: 5′-AATGCCGCAGTCGGCCGACCTG (AE4909), 5′-CAGGTCGGCCGACTGCGGCACTCGAGCTACTTCCCTGTTTAG (AE7005) and 5′-CTAAACAGGGAAGTAG (AE7006). Intasomes were assembled by mixing 180 μM MMTV IN with 52.9 μM bDNA (3.4:1 of IN:bDNA ratio) in 20 mM HEPES, pH 7.6, 600 mM NaCl, 2 mM DTT (240 μl final volume). After dialysis for 16 h at 4°C against 25 mM Tris–HCl, pH 7.4, 80 mM NaCl, 2 mM DTT, 25 μM ZnCl_2_, 10 mM CaCl_2_, the solution contained white precipitate that dissolved quickly upon NaCl addition to 250 mM final concentration. The sample was then incubated for 1 h on ice. The supernatant following centrifugation for 10 min at 20 000 g at 4°C was injected onto a Superdex 200 10/300 column equilibrated with 25 mM Tris–HCl, pH 7.4, 200 mM NaCl, 2 mM DTT, 25 μM ZnCl_2_, 10 mM CaCl_2_. Intasome-containing peak fractions (Supplementary Figure S1) were pooled and concentrated using 10 kDa molecular weight cutoff concentrators. The sample was then flash-frozen and saved for cryo-EM grid preparation.

### Cryo-EM vitrification and data acquisition

Purified MMTV STC sample (0.5 mg/ml) was applied to R1.2/1.3 gold UltrAufoil grids, Au 400 mesh (Quantifoil), and cryo-EM grids were prepared by freezing using a manual plunger at 4°C. The grids were clipped and subsequently stored in liquid nitrogen for future data acquisition. Data were collected at the Scripps Research Institute Cryo-EM facility, La Jolla using a FEI Titan Krios (300 kV) microscope equipped with a Gatan K2 summit direct detector. The complex was imaged at 22 500× magnification and the pixel size was 1.31 Å. Movies were collected in counting mode with an electron dose rate of 3.3 electron per pixel per second. The defocus range was −1.3 to −3.0 μm. A total of 1578 movies of 100 frames/movie were collected. The data collection parameters are presented in Supplementary Table S1.

### Cryo-EM data processing

CryoSPARC ver2.4 was used for all data processing steps (Supplementary Figure S2) (38). Movies were imported and corrected for patch motion and patch contrast transfer function (CTF). Output exposures were curated manually and two data subsets were selected that consisted of 480 and 812 micrographs representing 2.74–4.0 Å and 4.01–10 Å estimated CTF fit resolution ranges, respectively. Initial particle picking was performed with blob picker using 80–170 Å particle diameter range, and particles were extracted with 256 pixel box size and 2D classified. Six good classes were selected and served as templates for subsequent template picker jobs. Template picker outputted 631 649 and 1 048 508 particles for the first and second subset, respectively. Again, extracted particles were used in respective 2D classification jobs and, upon curation of suboptimal particles, two stacks consisting of 105 899 and 75 339 particles were obtained. Combined particles were then subjected to a single 2D classification (181 238 particles) to 100 classes, using 40 online-EM iterations, 5 final full iterations, 500 batchsize per class, and Force Max over poses/shifts as ‘OFF’. At this point, two separate processing strategies were followed. The first strategy focused on obtaining a high-resolution map for a single intasome complex. Only classes that had clear features were selected, resulting in 50 196 particles for subsequent steps. *Ab-initio* reconstruction imposing C2 symmetry followed by homogenous refinement resulted in a 3.8 Å global resolution map [Fourier shell correlation (FSC) = 0.143)]. Global CTF refinement was subsequently performed, followed by homogenous refinement and non-uniform refinement, yielding the final map of 3.5 Å global resolution (FSC = 0.143). In the second round of processing, we focused on obtaining a high-resolution map for the MMTV STC multimers that were uncovered during the course of investigation. Additional classes were picked after the 2D classification of 181 238 particles, which yielded 86 379 particles for further processing. Subsequent steps of data processing followed the same refinement and reconstruction protocols as above, yet using C1 symmetry. Finally, a refined map was obtained at 3.8 Å global resolution (FSC = 0.143). Both maps were analyzed/validated using the 3DFSC server (39). DeepEMhancer (40) 0.13 was run within the COSMIC cryo-EM platform (41).

### Model building and refinement

Initial model building was accomplished by rigid-body fitting of the MMTV CSC structure (Protein Data Bank identification code (PDB ID) 3JCA) into the EM map in Chimera 1.14 by ‘Fit in Map’ tool (42). Unmodeled protein and DNA residues were interactively built in Coot 0.9.4.1 (43) and the structure underwent a few iterative cycles of manual model re-building and real-space refinement in Phenix (44,45). Ramachandran and secondary structure restraints were applied. This model encompassed the nucleic acids and the core region of the MMTV STC intasome. To model the full octameric intasome, we first rigid-body docked the flanking IN dimers (PDB ID: 5CZ2) into the map. The flanking IN dimers were then refined into the density independently of the core region. The density connecting the flanking dimers and the core was evident, but broken, and therefore a model was not derived for the linker regions, but a connector was placed between the flanking subunits and the CTD domains within the core region. The final model accounts for the complete octameric MMTV STC intasome with connections for the linker regions, deposited as PDB ID 7USF. We also modeled the di-intasome stacked from two STCs, resolved to 3.8 Å global resolution. For this model, MMTV STC intasomes from above were rigid body docked into the EM map in Chimera, and any components that were not represented by density were removed. It was also necessary to rigid-body dock the NTD:CCD dimer into the flanking regions of the map. The whole assembly was then subjected to a refinement in Phenix, similarly as described above. This model accounts for the double MMTV STC higher-order assembly, deposited as PDB ID 7UT1. The quality of all modeling results was validated using Molprobity metrics (46), available in Phenix or as a standalone web server. All images were generated using UCSF Chimera (42). The alignment in Supplementary Figure S10 was made using ESPript 3.0 (47).

### Analysis of IN-DNA interactions and DNA bending

Structural analyses for the IN-DNA interactions were performed using the DNAProDB web server (48,49), available at https://dnaprodb.usc.edu/. Interactions were based on default parameters suggested in the publication (48,49), and on the DNAProDB server, which includes cutoff distances of 3.9 Å for Van der Waals as well as for donor—acceptor H-bonds (wherein donor indicates the heavier atom). Figures were generated based on the output of the program. Analyses of DNA bending were performed using the 3DNA web server (50), available at http://web.x3dna.org/. The widths of the major groove, the rise and roll of bp steps, the form of the DNA, among other parameters were outputted from the program and mapped onto the structure.

In addition to the PFV STC structure with naked tDNA, we analyzed the PFV STC-nucleosome complex (PDB ID: 6RNY) (51). While pronounced A-ness was observed at one site of vDNA/tDNA joining, this observation did not hold for the second site. However, the cryo-EM map used to derive the STC-nucleosome model was insufficiently resolved at the nucleosomal tDNA to properly position and distinguish DNA nucleotide parameters. At low resolution, modelling and refinement restraints play a significant role to dictate DNA conformation. Thus, resolution limitations hindered our ability to make satisfactory conclusions about tDNA conformation in the nucleosome-engaged STC structure.

### IN enzyme assays

IN 3′-processing and DNA strand transfer activities were determined essentially as previously described using oligonucleotide mimics of the viral U5 end (34). For 3′-processing assays, the 5′ end of the viral plus-strand (AE7918; 5′-GTGACCCTCAGGTCGGCCGACTGCGGCA/TT; slash denotes position of IN-mediated hydrolysis) was labeled using γ^32^P-ATP and T4 polynucleotide kinase for 45 min under conditions recommended by the manufacturer (New England Biolabs). EDTA was added to the final concentration of 14.3 mM, after which the mixture was incubated at 85°C for 15 min to heat-inactivate the kinase. The complementary minus strand (AE7919; 5′-AATGCCGCAGTCGGCCGACCTGAGGGTCAC) was annealed to the plus-strand in the presence of 0.1 M NaCl by heating at 85°C for 2 min, followed by stepwise cooling to 20°C at the rate of 1°C/min over 65 min. The labeled DNA was separated from free nucleotide using a P6 spin column (Bio-Rad) equilibrated in 20 mM NaCl, 10 mM Tris–HCl, pH 8.0, 0.1 mM EDTA.

IN 3′-processing reactions (16 μl) contained 25 mM MOPS, pH 7.2, 10 mM DTT, 5 μM ZnSO_4_, 10 mM MgCl_2_, 15 nM labeled vDNA, and 0.8 μM IN. Following incubation at 37°C for 1 h, reactions were terminated by adding an equal volume of sequencing gel sample buffer (95% formamide, 0.03% xylene cyanol FF, 0.03% bromophenol, 10 mM EDTA, pH 8.0) and heating at 100°C for 2 min. Reaction aliquots were fractionated through 15% polyacrylamide DNA sequencing gels. Wet gels exposed to phosphor screens were imaged using a Typhoon Variable Mode Imager (Cytiva). 3′ Processing activity was quantified using ImageQuant TL v8.2.0.0 software as the percent of 30-mer substrate DNA converted into 28 nt reaction product.

IN strand transfer activities were assessed using pGEM-3 plasmid as tDNA essentially as previously described (14). Reaction aliquots were fractionated through agarose gels, and gels stained with ethidium bromide were imaged using a ChemiDoc MP imager (Bio-Rad). IN mutant integration activities were quantified using Fiji software as percent product formation versus the WT enzyme.

### Virus production and infection

HEK293T cells, which were used to produce MMTV by plasmid DNA transfection and also as target cells for infection assays, were propagated at 37°C in Dulbecco's modified Eagle's media supplemented to contain 10% fetal bovine serum, 100 IU/ml penicillin, and 100 μg/ml streptomycin (DMEM) in humidified incubators in the presence of 5% CO_2_. Viruses were produced by co-transfecting cells seeded in 15 cm tissue culture dishes with pRRpCLucWPRE25, pCMVgpRRE17, pRSV-Rev, and pCG-VSV-G plasmid DNAs mixed at the ratio of 6:5:3.6:1.1, respectively (30 μg total plasmid DNA), using PolyJet DNA transfection reagent (SignaGen Laboratories). After 48 h, virus-containing supernatant clarified at 600 × g for 5 min was filtered by gravity through 0.45 μm syringe filters. Aliquots, which were stored at −80°C for 6–12 months, were thawed once for infection assays. MMTV concentration in mU/ml was determined using a TaqMan-based product-enhanced exogenous reverse transcriptase (RT) assay as described (16).

Cells (10^5^) were infected in duplicate with 5 mU of WT and IN mutant MMTV RT activity in 24-well-plates for 6–8 h, after which the virus-containing media was replaced with fresh DMEM. Cells were processed for luciferase assays at 48 h post-infection as described (52). Luciferase activity was normalized to the amount of protein in the cell extracts as described (52).

Levels of MMTV capsid and IN proteins in virus lysates were determined by immunoblotting essentially as previously described (52). Primary antibodies were procured from Rockland Immunochemicals (CA, catalog number 100-401-P12) or Thermo Fisher (IN, catalog number HAB2110A, which was affinity-purified sera produced from rabbits inoculated with purified MMTV IN). Horseradish peroxidase (HRP)-conjugated secondary anti-rabbit IgG was from Dako. To enhance IN detection, IN was immunoprecipitated from MMTV lysates using HAB2110 versus control IgG (Thermo Fisher catalog number 02–6102) antibodies prior to SDS-PAGE, and these membranes were probed with biotinylated HAB2110 antibodies that had been labelled using the One-Step Antibody Biotinylation Kit (catalog number 130-093-385) as recommended by the manufacturer (Miltenyi Biotec). Washed membranes were then incubated with streptavidin-HRP (Thermo Fisher) diluted 1:60 000 in a 5% solution of bovine serum albumin. Immunoblot signals developed using SuperSignal West Pico PLUS Chemiluminescent Substrate as recommended by the manufacturer (Thermo Fisher) were recorded on the ChemiDoc MP imager. Capsid and IN levels in IN mutant virions were normalized to respective signals detected in WT virions.

### MMTV integration site analyses

Genomic DNA from MMTV-infected cells was harvested 5 days after the start of infection. Manipulations of DNA for LM-PCR library generation for Illumina sequencing, and downstream bioinformatics analyses of resulting Illumina reads and the mapping of filtered reads to human genomic DNA annotations, followed methodologies previously established for other retroviruses (52–54). In brief, genomic DNA (10 μg) from duplicate sets of infections was digested with MseI and PstI-HF restriction endonucleases (New England Biolabs) overnight. Fragmented DNA was subsequently ligated to asymmetric DNA linkers containing compatible 5'-TA overhangs (see Supplementary Table S2 for linker and PCR primer sequences used for LM-PCR library generation). MMTV U5-host DNA junction sequences were preferentially amplified via two rounds of PCR. Primers for the first round included a U5 DNA-specific LTR primer and either of two different linker-specific megaprimers that harbored sequences compatible with Illumina cluster formation and sequencing (Supplementary Table S2). Second round PCR primers included the same linker-specific megaprimer and a nested LTR megaprimer that in addition to Illumina cluster and sequencing sequences contained a unique 6 nucleotide barcode (Supplementary Table S2). LM-PCR products were subjected to 150 bp paired end Illumina HiSeq sequencing at Genewiz.

Following the processing of raw Illumina sequence reads, filtered integration sites from duplicate infections were combined and mapped to human genome build hg38 as previously described (52–54). Fisher's exact test was used to assess the statistical relevance of WT and IN mutant viral integration frequencies into genes, speckle-associated domains (SPADs), nearby transcriptional start sites, and lamina-associated domains (LADs). Wilcoxon rank sum test was used to assess statistical relevance of gene dense region targeting (results of statistical analyses are tabulated in Supplementary Table S3). Sequences proximal to sites of WT and IN mutant vDNA integration were visualized using WebLogo (55) and percent YR/RY usage (8,26,27) as described (8).

### Free energy profiles associated with B-to-A transition in genomic DNA around retroviral integration sites

Sequence-dependent free energies for the B-to-A transition tabulated for all trinucleotide combinations in Tolstorukov *et al.* (56) were used to generate plots for the free energy associated with the B-to-A transition in genomic DNA. In brief, *in cellulo* or *in vitro* integration sites for MMTV (18 492 unique cellular sites, this work), PFV (4 407 926 unique *in vitro* sites (57); 226 146 unique cellular sites (58)), MVV (411 720 unique cellular sites (19); 327 851 unique *in vitro* sites (15)), HIV-1 (43 232 unique cellular sites (52,59)), HTLV-1 (235 139 unique cellular sites (60)) and MLV (264 353 unique cellular sites, (52)) were extracted from human genome assemblies using fastafrombed program from BEDtools suite (61). A Δ*G*_B→A_ value was then assigned for each trinucleotide in the aligned genomic DNA sequences. A running tab was kept for the sum and average Δ*G*_B→A_ value at each corresponding position, and average values were plotted across aligned bp steps.

The above-referenced integration site datasets were derived from experiments that sequenced only one of the two integrated provirus-host junctions. The site of genomic DNA insertion of the other vDNA end was inferred from the known TSD of the corresponding retroviral species. Although several hundred virus-host junctions derived from one end of RSV/avian sarcoma-leukosis virus (ASLV) proviruses were also available (62–64), we excluded these from our analyses because RSV/ASLV integration yields a mixture of 5 and 6 bp TSDs (65,66). Mixed TSDs preclude clear A-philicity analysis of integration sites derived from only one end of proviral DNA.

## RESULTS

### MMTV STC assembly and cryo-EM analysis

**Figure 1.**
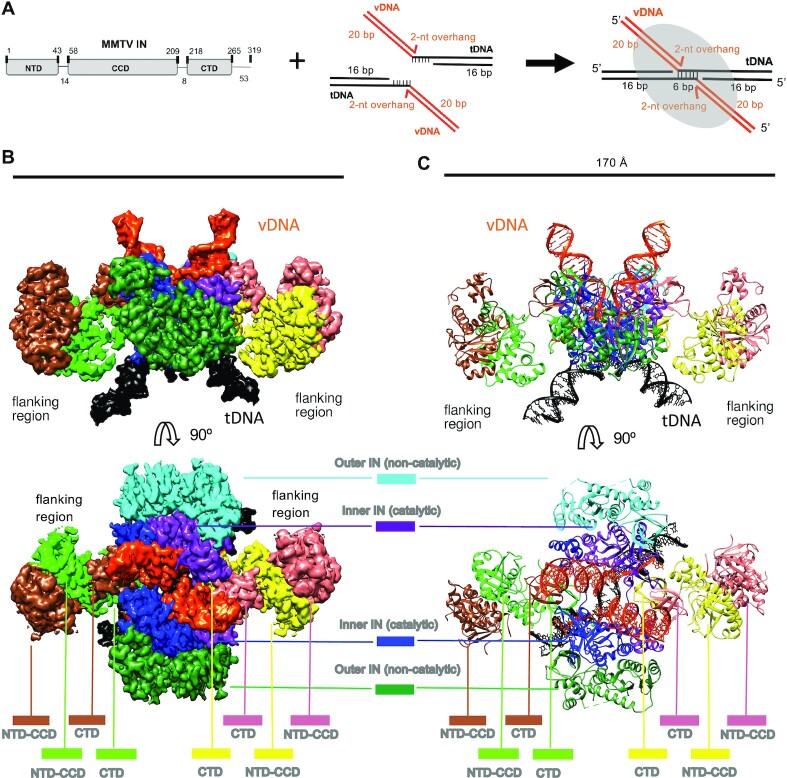
Architecture of the MMTV STC intasome. (**A**) Schematic showing MMTV IN and bDNA substrate used for STC intasome assembly. (**B**) Side (above) and top (below) views of the cryo-EM reconstruction. The displayed map is the output of DeepEMhancer (40). (**C**) Model of the octameric MMTV STC structure. Both the map and model are colored by IN protomer/DNA fragment; vDNA/tDNA colors match the panel A schematic.

PFV STCs assembled with branched DNA (bDNA) mimicking the concerted integration product are indistinguishable from those resulting from IN-mediated strand transfer (8,67). To recapitulate the TSD observed in MMTV infected cells (68), we assembled MMTV STCs using a bDNA that harbored two vDNA ends covalently joined to opposing tDNA 5'-phosphates separated by 6 bp (Figure 1A, Supplementary Figure S1A). The tDNA portion of the bDNA was palindromic, with invariant CA-3′ vDNA ends linked to 5′-CTCGAG sequences (Supplementary Figure S1A, B). We interchangeably refer to this region as sites of vDNA joining or tDNA cleavage. The 5' tDNA cytosines at the positions of vDNA joining are by convention assigned base zero. As previously done for the MMTV CSC, Ca^2+^, which can enhance specific IN-vDNA interactions without supporting IN catalytic function (69), was included to enhance STC assembly. Assembly reactions were subjected to size exclusion chromatography, which revealed peaks corresponding to free IN and bDNA, as well as a higher-order species with *A*_260_/*A*_280_ ratio indicative of a nucleoprotein complex (Supplementary Figure S1C).

We vitrified pooled column fractions containing STC intasomes on UltraAuFoil gold grids (70) and collected 1578 cryo-EM movies using the Titan Krios microscope and the K2 direct electron detector (Supplementary Table S1). We then subjected the resulting dataset to an iterative 2D and 3D classification analysis, which yielded a final stack of 50 196 particles and a map resolved globally to 3.5 Å (Supplementary Figures S2 and S3). The central portion containing the two catalytically-competent MMTV IN subunits was resolved to ∼3 Å. Overall, the cryo-EM map demonstrated clear density for most IN side chains in the intasome core region, including those that interact with vDNA and tDNA, which facilitated derivation of an atomic model. The comparatively flexible flanking regions of the STC were resolved to lower resolution, but the respective IN NTD-CCD dimers could nonetheless be refined based on a 2.7 Å X-ray structure [PDB ID: 5CZ2 (14)]. The final model was consistent with the cryo-EM map and was characterized by good geometry statistics (Supplementary Table S1 and Supplemental Figure S3).

### Architecture of the octameric MMTV STC intasome

As observed for the MMTV CSC intasome (14), the STC complex comprises eight IN molecules (Figure 1B-C). The overall architecture can be subdivided into a central core region and two flanking regions, one on each side of the core. Each flanking region comprises an IN NTD-CCD dimer that donates a pair of CTDs to the central core. Consequently, the flanking regions are loosely tethered to the core via ∼8-residue flexible CCD-CTD linkers. As also seen for the CSC (14), the flanking regions of the STC are conformationally flexible with only minor, if any, stabilization imparted by the tDNA. The central region includes four CTDs emanating from the flanking dimers (two from each side) and four IN protomers in which all domains are constrained to the core region. The latter can be subdivided into two inner INs that perform the catalytic cleavage reactions, which in the STC contact both vDNA and tDNA, and two outer INs that are not involved in catalysis. Collectively, the architecture captures densities for all IN domains for each protomer within the STC. Experimental density was not observed for amino acids beyond Glu269 for any of the IN protomers, indicating that C-terminal residues spanning from Glu270 to Pro319 are disordered. A comparison of the previously determined CSC and current STC structures corroborated the nearly identical overall arrangement of respective IN domains and vDNA molecules, with a root mean square deviation of 0.75 Å for Cα atoms of the 1104 aligned protein residues (Supplementary Figure S4). The presence of tDNA within the STC complex accounts for the major difference between the two structures.

During image analysis, we noted additional particles that could not be attributed to octameric STCs. Further investigation through iterative sub-classification revealed multiple interacting STCs, the structure of which was independently refined to ∼4 Å resolution (Supplementary Figure S5). Rigid body docking supported positioning of up to three STCs, one on either side of a central nucleating complex, primarily held together through interactions mediated by flanking NTD-CCD dimers (Supplementary Figure S6A–D), tDNAs and CCDs (Supplementary Figure S6E), and adjacent CCDs (Supplementary Figure S6F, G). Because this higher-order STC assembly lacks obvious biological relevance, we will not comment on it further.

### MMTV IN induces a pronounced tDNA bend and A-form DNA around the cleavage sites

**Figure 2.**
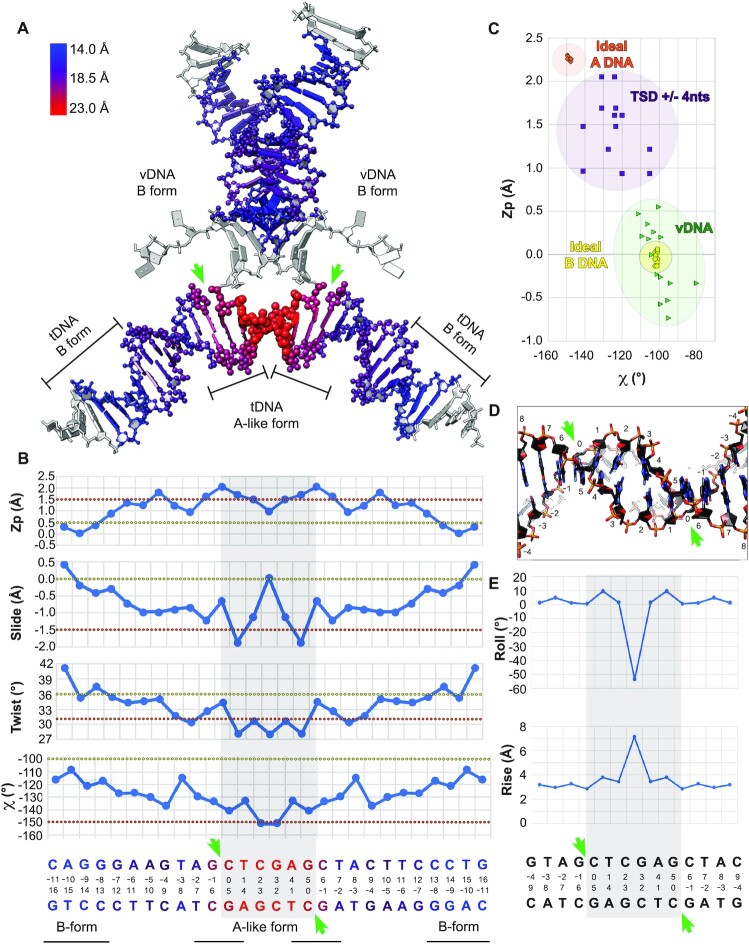
Local deformation and A-form tDNA. (**A**) Model of the MMTV STC vDNA and tDNA nucleic acids. Coloring corresponds to the width of the major groove, in Å. DNA forms within local regions are indicated. (**B**) Plots corresponding to *Z*_p_, slide, twist, and χ for the indicated regions of tDNA. Each bp corresponds to a box, whereas a bp step, used for *Z*_p_, slide and twist calculations, corresponds to an edge. The tDNA sequence is shown below, colored as in (A), with 6 bp tDNA cleavage site highlighted by gray shade. Values typical of A- versus B-DNA in the different panels are indicated by orange and yellow dotted lines, respectively. (**C**) Plot of *Z*_p_ versus χ for the vDNA and for the central fourteen bp of tDNA (6 bp cleavage site ± 4 abutting bp on each side). For comparison, ideal A- and B-form DNA are plotted. (**D**) Close-up of the target site, shown from below. (**E**) Plots of the roll and rise for the bp steps within and around the vicinity of the tDNA cleavage sites. In all panels, green arrows demarcate the cleavage sites.

The high-resolution information evident in the map allowed us to elucidate the details of tDNA conformation and recognition by MMTV IN. A striking feature of the MMTV STC is the pronounced bend and characteristic deformation in the portion of the tDNA that surrounds vDNA joining sites (Figure 2A). To quantify the deformation, we analyzed DNA parameters using the program 3DNA (50). In comparison to the vDNA or to the peripheral regions of tDNA, which maintain ∼16 Å phosphate-phosphate distances in the major grooves, the tDNA major groove begins to noticeably widen at bp positions –1/+6, reaching a maximum distance of ∼22 Å within the central two dinucleotides of the cleavage site. The entire 6-bp cleavage site is characterized by a major groove width of ≥20 Å (Figure 2A).

The tDNA configuration in and around the 6-bp cleavage site exhibits multiple characteristics of A-form DNA. Several structural features distinguish A- and B-forms of DNA. The Z_p_ parameter reflects the mean z-coordinates of the backbone phosphorus atoms with respect to the reference frame of an individual dinucleotide dimer; *Z*_p_ is ≳1.5 Å for A-DNA and ≲0.5 Å for B-DNA. The Slide parameter reflects the relative motion of two stacked bp along their long axes; Slide is ≲−1.5 Å for A-DNA and ≳0.0 Å for B-DNA. The Twist parameter reflects the rotation, from the local perspective, of two stacked bp about an axis perpendicular to the mean plane; Twist is ∼31° for A-DNA and ∼36° for B-DNA. Lastly, the χ parameter reflects the backbone torsion angle between the sugar and base; χ is ∼−150° for A-DNA and ∼−100° for B-DNA. While bp steps are used to calculate *Z*_p_, slide and twist, individual bp measures underlie the χ parameter. Across these metrics, there is a clear trend toward the A-like conformation surrounding the tDNA cleavage sites (Figure 2B). A plot of *Z*_p_ versus χ best distinguishes A- and B-form DNA, as *Z*_p_ values relate strongly to the mean glycosyl torsion parameter χ (71). Although such analyses distinguish the DNA forms, clear-cut cases of A-DNA are rare, and most DNA conformations tend to fall in a range. Indeed, in such a plot, the base steps in and around the tDNA cleavage sites adopt A-like characteristics that are readily distinguishable from the base steps for vDNA, which approach ideal B-form (Figure 2C). Within the 6-bp cleavage site, the central dinucleotides at position 2–3 are particularly deformed, characterized by a ∼50° negative roll and ∼6 Å rise (Figure 2D, E). The deformation in the center of the target site likely accommodates the gradual transition from B- to A-DNA along each tDNA strand and ensures optimal positioning of the two scissile phosphodiester bonds at the enzyme active sites for IN-mediated strand transfer activity.

### Interactions between MMTV IN and nucleic acids

**Figure 3.**
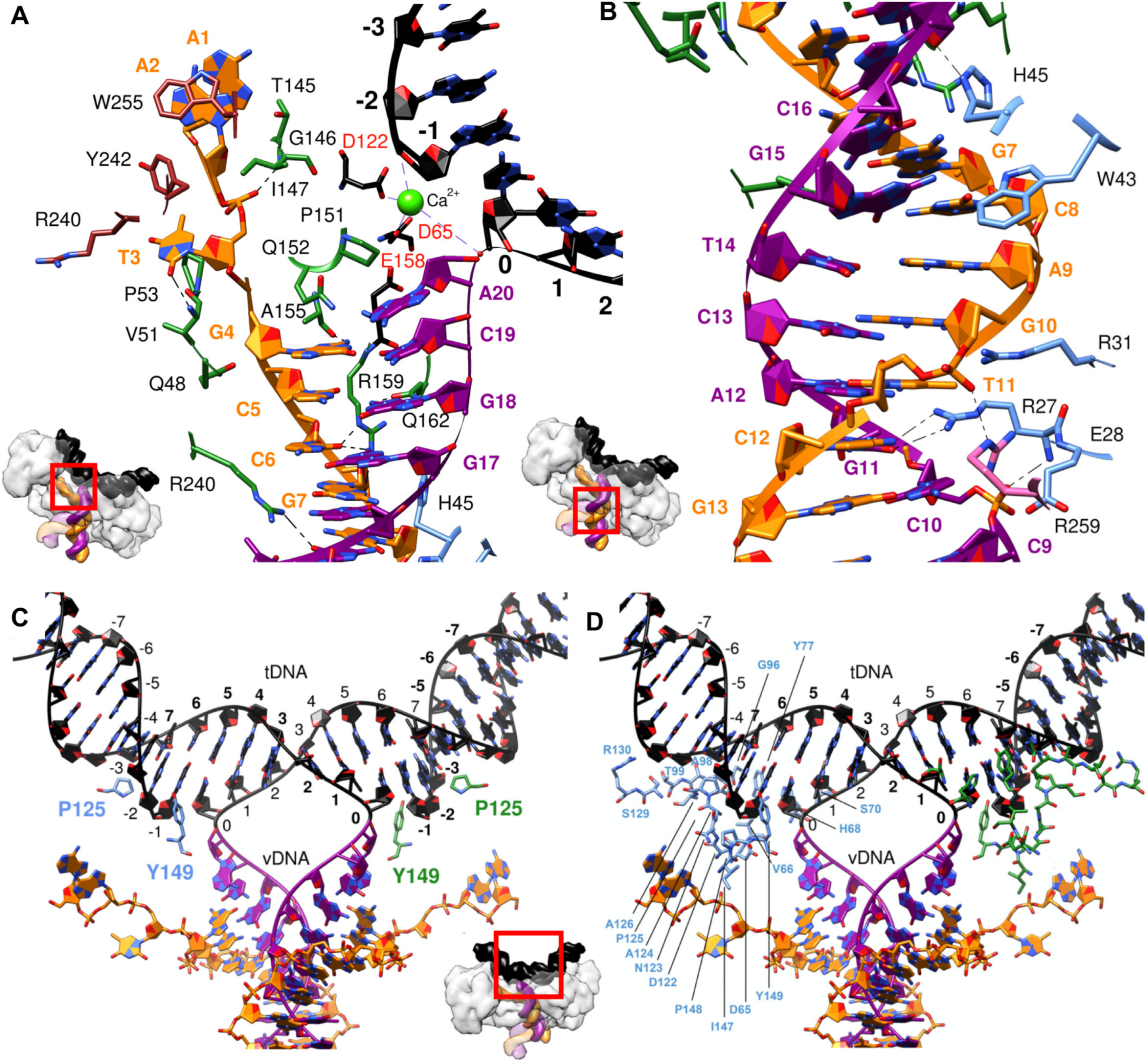
Structural overview of select interactions between IN and nucleic acids within the MMTV STC. (**A, B**) IN–vDNA interactions either (**A**) proximal to or (**B**) distal from the MMTV IN active site. (**C**, **D**) IN–tDNA contacts, including (**C**) base-specific interactions (Pro125) and/or sidechain insertions into tDNA major/minor grooves (Tyr149) and (**D**) backbone interactions. In all panels, nucleotides are labeled in bold, amino acid side chains are colored by their respective protein chains, the non-transferred vDNA strand is in orange, the transferred vDNA strand is in magenta, tDNA is in black, and catalytic triad DDE residues Asp65, Asp122 and Glu158 are labeled in red. Insets show the regions of the STC enlarged (red boxes) within separate panels.

A comprehensive analysis of nucleoprotein interfaces identified IN amino acid residues that interact with the bDNA substrate. We present these results in two different figures to distinguish base-specific interactions (Supplementary Figure S7, which also includes sidechains that interact with DNA major or minor grooves) from contacts with the sugar-phosphate backbone (Supplementary Figure S8). Six different IN chains within the octamer, and all three IN domains, contact distinct vDNA regions (Figure 3A, B). IN protomers contact most of the ∼10 bases of vDNA adjacent to the tDNA cleavage sites, including the unpaired 5′-AA overhangs of the non-transferred vDNA strand. IN CTD residue Trp255 mediates a π−π−π stacking network between its aromatic side chain with the 5′-AA overhang. NTD−CCD linker residues Pro53, Val51, and Gln48 abut T3 and G4 bases of the non-transferred vDNA strand (Figure 3A). However, the most extensive contacts with vDNA are mediated by the CCDs of the inner IN protomers. At the IN active site, a 3_10_ helix containing residues Pro151, Gln152 and Ala155 cradles the vDNA bases immediately preceding the cut site. Arg159, which abuts Glu158 of the catalytic DDE triad, inserts into the minor groove of vDNA upstream of the invariant CA dinucleotide (Figure 3A). Other nearby residues that insert into the minor groove include Gln162, His45 and Trp43 (Figure 3A, B). On the opposite side of the vDNA duplex, multiple residues protrude into the vDNA major groove, including Arg240 from an adjacent CTD, Arg31 and Arg27 from the proximal NTD, and Arg259 from a CTD of a distinct chain (Figure 3A, B and Supplementary Figure S7). Collectively, the structure reveals an array of IN-base interactions that likely confer specificity for the extremities of the MMTV LTRs.

In contrast to the comparatively extensive base-specific vDNA interfaces, IN interacts with only three nucleobases of tDNA, immediately adjacent to either side of the cleavage sites. CCD residue Pro125 makes base-specific contacts with tDNA bases at positions −3/+8, and Tyr149 inserts into the tDNA major groove, contacting bp positions −1/+6 and −2/+7 (Figure 3C). The relative dearth of base-specific tDNA contacts is consistent with only very modest tDNA sequence preferences observed at sites of retroviral integration (22–28). Most IN residues that interact with tDNA accordingly contact the sugar-phosphate backbone. A 5-bp stretch downstream from the cleavage site along the joined strand is contacted by an array of IN residues, including Tyr77, Gly96, Thr99, Pro125, Ala126, Ser129, and Arg130 (Figure 3D and Supplementary Figure S8). This configuration—in which there are pronounced IN-tDNA contacts along the cleaved strand distal from the points of cleavage—suggests that IN−tDNA backbone interactions exert force on the system that is translated along the tDNA lever arm to bend and distort the target site to facilitate integration.

Prior to this work, intasome STC structures were derived for PFV (8), RSV (12), HIV-1 (17), HTLV-1 (10) and MVV (19). Although backbone-specific contacts between IN and tDNA were previously reported (8), their extent and orientation of the contacts with respect to the cleavage site were not fully appreciated. To gain further insight, we analyzed all known STC intasome structures for IN-tDNA contacts, which resulted in the following observations (Supplementary Figure S9): (i) base-specific tDNA contacts are generally infrequent relative to backbone-specific contacts; (ii) in comparison to backbone interactions upstream of the sites of tDNA cleavage, there are always more interactions downstream, and they extend further out, up to 9−12 bases along the joined strand; (iii) central regions interior to the cleavage sites (gray shades in Supplementary Figure S9) are generally devoid of both backbone- and base-specific interactions, suggesting the requirement for flexibility. Thus, IN−tDNA backbone contacts are architecturally conserved across retroviral STCs and likely help to induce tDNA distortions necessary for IN strand transfer activity.

There are comparatively few conserved residues that contact specific tDNA bases or reach into a tDNA groove. Contacts between residues analogous to MMTV IN Pro125 and tDNA are observed across retroviral STCs, and Pro125 is concordantly conserved across *Retroviridae* as a comparatively small residue (e.g. Ala188 in PFV, Ser119 in HIV-1, Pro123 in HTLV-1, Pro121 in MVV and Ser124 in RSV). A second notable tDNA-interacting MMTV IN residue is Tyr149, which lies in a short loop immediately preceding the CCD 3_10_ helix and inserts its sidechain into the tDNA major groove at the cleavage site, making contacts with the backbone (Figure 3C). Pro125 and Tyr149 reside on opposite sides of the phosphate backbone, immediately adjoining each of the two tDNA cleavage sites, clasping the cleaved tDNA strand (Supplementary Figure S10A, B). The configuration of MMTV IN Pro125 is a shared feature of all retroviral STCs, where analogous residues pack against the tDNA minor groove in close vicinity of the scissile phosphodiesters irrespective of the 4–6 bp spacing between the sites of vDNA joining (Supplementary Figure S10A). The presence and/or orientation of residues equivalent to MMTV IN Tyr149, by contrast, vary across retroviral STC structures. Most INs harbor Tyr at the position analogous to 149 in MMTV IN, whereas the INs of the ungulate lentiviruses, MVV and caprine anemia encephalitis virus, harbor Trp at the equivalent positions. By contrast, RSV IN features a Gly at this position and accordingly stands out as the only IN among the solved structures that does not use a bulky hydrophobic sidechain to contact tDNA (Supplementary Figure S10C). A third comparatively conserved tDNA contact across retroviral intasomes originates from a charged residue within the CTD β1-β2 loop (Arg329 in PFV, Arg231 in HIV-1, Arg231 in MVV and Glu229 in RSV) (Supplementary Figure S10A–D). Neither MMTV nor HTLV-1 IN harbor a similarly charged residue at this position, which likely accounts for the lack of a CTD β1-β2 loop sidechain contact to tDNA in these structures. In these cases, hydrophobic CTD β1–β2 loop residues may help to stabilize Tyr147/Tyr149 interactions with tDNA major grooves (Supplementary Figure S10B).

### Biochemical activities of IN mutant proteins

We next examined how perturbation of select MMTV IN residues that were observed to mediate key interactions in our structure affect IN activity and tDNA selectivity. To assess IN–vDNA interactions, we targeted Trp255, which interacts with vDNA near the sites of vDNA joining, as well as Arg159, Arg27 and Arg31, which interact with vDNA distal from the tDNA (Figure 3A, B). We generated bacterial expression vectors for IN missense mutants R159E, W255A and R27A/R31A (RRAA), and purified the proteins following their expression in *Escherichia coli*. To assess IN-tDNA interactions, we expressed and purified P125T, P125D and Y149G IN mutant proteins.

Two additional mutants, D223A and D223R, were generated to test the importance of the flanking IN dimers in MMTV integration. Our previous work hinted at the importance of the flanking IN dimers for IN activity through mutagenesis of residue Arg240 (14). Arg240 from a flanking IN protomer makes a prominent salt-bridge with Asp223 from a core protomer, and we previously assessed the *in vitro* activity of IN mutant R240E. This mutant IN was however defective for strand transfer activity, likely due to the involvement of Arg240 in multiple IN–IN and IN–vDNA interactions [Figure 3A and (14)]. Herein, we targeted Asp223, the other partner of the intermolecular salt bridge, with the hope to sidestep the pleiotropic effects observed previously with R240E mutant IN.

**Figure 4.**
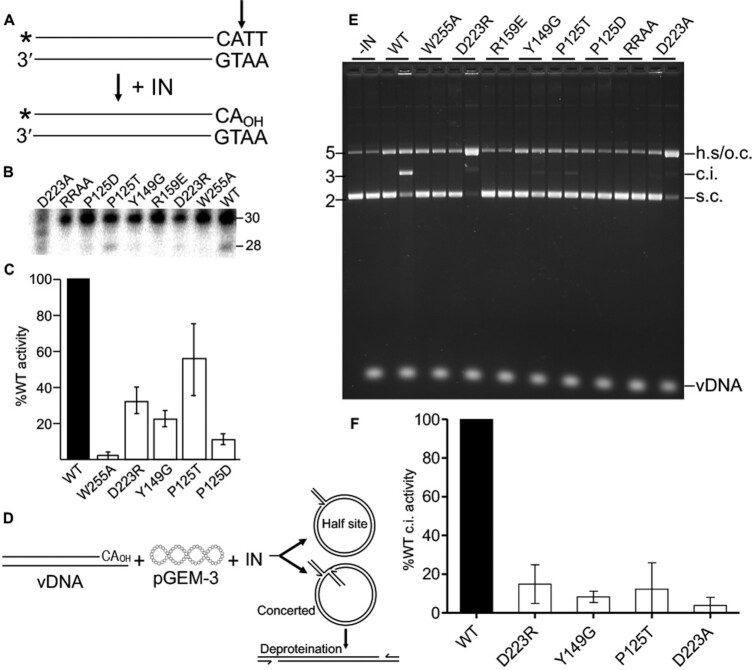
3′-Processing and strand transfer activities of IN mutant proteins. (**A**) 3′-Processing assay schematic. U5 substrate (30 bp) was labeled with ^32^P (marked *) at the 5′ end of the transferred strand (cleavage site marked by vertical arrow). (**B**) Representative 3′-processing gel image. Positions of 30 base substrate and 28 base 3′-processing product are noted; the assayed IN proteins are denoted above the phosphor image (RRAA = R27A/R31A). (**C**) 3′-Processing results of IN mutant proteins whose activities were greater than background (average ± standard error for *n* = 3 experiments). Results were normalized to WT IN activity, which across experiments converted 12.2% ± 3.4% of substrate to product. D223A activity was not assessed due to apparent contaminating exonuclease activity in the IN prep. (**D**) Schematic of strand transfer assay. Integration of precleaved vDNA into supercoiled (s.c.) pGEM-3 can yield a half site (h.s.) integration product that comigrates with open circular (o.c.) pGEM-3 on agarose gels or a concerted integration (c.i.) product that migrates marginally slower than linearized plasmid DNA. (**E**) Representative agarose gel image. Positions of DNA standards, shown to the left, are in kb; positions of substrate and product DNAs are at right. The noted IN (or buffer control; -IN) was reacted with pGEM-3 in the absence (first lane of two-lane pair) or in the presence of vDNA; the minus-vDNA samples control for non-specific endonuclease activity that may be present in the IN prep. While starting pGEM-3 was predominantly supercoiled, note a modicum of o.c. in -vDNA lanes. (**F**) Quantification of IN mutant protein c.i. activities (for mutants whose activities were greater than background; average ± standard error for *n* = 3, normalized to WT activity).

The mutant proteins were analyzed alongside WT IN for 3′-processing and strand transfer activities using double-stranded oligonucleotide mimics of the U5 vDNA end (Figure 4). For 3′-processing, the 5′-end of the transferred vDNA strand was labelled with ^32^P; 3′-processing by IN liberates the terminal TT dinucleotide, yielding a labeled 28-mer strand that is readily resolved from the substrate 30-nt strand by denaturing polyacrylamide gel electrophoresis (Figure 4A, B). Across replicate experiments, WT IN converted approximately 12% of the vDNA substrate to the 3′-processed reaction product (Figure 4B). The activities of the IN mutants were normalized to the level of activity of WT IN. IN mutant proteins P125D, P125T, Y149G and D223R displayed partial 3′-processing activity, which ranged from about 10–60% of WT IN activity across replicate experiments. While W255A was minimally active, IN mutants RRAA and R159E failed to support detectable levels of IN 3′-processing activity (Figure 4B, C). These data highlight the importance of vDNA contacts mediated via IN residues Arg159 and Arg27/Arg31 for IN function.

IN strand transfer reactions utilized pre-processed vDNA and supercoiled pGEM-3 plasmid as tDNA. Under these conditions, concerted integration (c.i.) of two vDNA strands linearizes pGEM-3, which, after deproteination, yields an integration product that migrates in an agarose gel near the position of linear pGEM-3 (Figure 4D, E). In case IN supports the integration of only one instead of two vDNA ends, the resulting half-site (h.s.) integration product after deproteination would co-migrate with open-circular plasmid DNA. To ensure evaluation of vDNA-specific nuclease activity, paired reactions were conducted in the presence or absence of vDNA. WT IN supported robust c.i. activity without any evidence for h.s. integration activity (Figure 4E). As above, IN mutant c.i. activity was normalized to the level of WT IN activity. Consistent with their inabilities to effectively process vDNA ends, IN mutants W255A, R159E and RRAA were defective for IN strand transfer activity. IN mutants D223R, D223A, Y149G and P125T displayed marginal c.i. activities that ranged from just a few percent to ∼10% of WT IN activity across replicate experiments (Figure 4E, F). At the same time, IN mutant D223A and D223R h.s. integration activities were increased ∼30- to 40-fold over the background open-circular plasmid signal present in the WT IN reaction lane. None of the other tested IN mutants displayed detectable levels of vDNA-dependent h.s. integration activity (Figure 4E).

### Infectivity and integration site distributions of MMTV IN mutant viruses

IN mutant proteins that displayed partial 3′-processing and strand transfer activities *in vitro* were next evaluated under conditions of MMTV infection. For this, we leveraged a previously described single-round infection system where MMTV structural proteins and enzymes, expressed from a Gag-Pol plasmid, encapsulate an MMTV genome that is engineered to express a reporter gene following virus infection of a target cell (35). To streamline quantification and enhance detection of viral infection levels, we substituted the original reporter gene encoding green fluorescent protein (35) for firefly luciferase (72). We included two control viruses in addition to test IN mutant P125D/T, Y149G and D223A/R constructs. The D122N control harbored the conservative substitution of Asn for the second Asp residue of the DDE catalytic triad. Stop codons were introduced into the *pol* gene after the RT coding portion to yield the IN-deletion control virus, delIN. Viruses were pseudotyped by co-transfection with an additional plasmid that expressed the vesicular stomatitis virus G glycoprotein (VSV-G). Levels of WT and IN mutant viruses in transfected cell supernatants were assessed for virus-associated RT activity, and normalized levels of WT and IN mutant RT activity were utilized in downstream virus assays.

**Figure 5.**
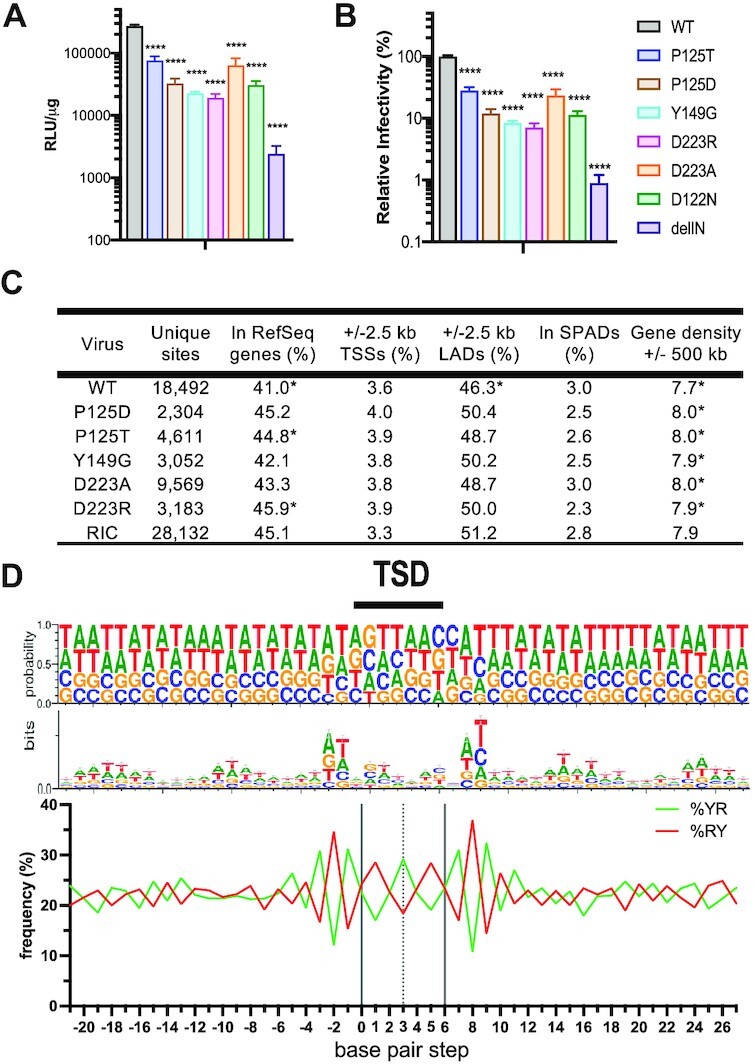
WT and IN mutant MMTV infection and integration targeting profiles. (**A**) Viral infectivities in relative light units (RLUs) normalized to levels of total protein in cell extracts. (**B**) Same as in panel A, regraphed to normalize mutant IN infectivity to WT MMTV, which was set at 100%. Results are average ± standard deviation (s.t.d.) for *n* = 3 independent infection experiments, with each experiment conducted in duplicate. Statistical difference versus WT MMTV (****P< 0.001) was determined by one-way ANOVA. (delIN = IN deletion control virus). (**C**) Integration targeting frequencies. Statistically different values (*, *P*< 0.01) for WT versus computer-generated RIC values and for mutants versus WT were determined by Fisher's exact test or, in the case of gene density, Wilcoxon rank sum test. See Supplementary Table S3 for *P* values of expanded pairwise comparisons. LADs and SPADs refer to lamina-associated domains and speckle-associated domains, respectively. The noted integration sites were compiled from two independent infection experiments. (**D**) Base preferences at sites of WT MMTV integration. The upper portion shows the probability of base usage for a 50 bp stretch centered on the TSD. The middle and lower portions display these results via base logo (middle row) and percent YR versus RY dinucleotide (lower) usage.

Immunoblotting virus lysates revealed similar levels of viral capsid protein contained within the different WT and IN mutant virus preparations (Supplementary Figure S11A). To enhance detection of packaged IN proteins, viral lysates were concentrated by immunoprecipitation prior to immunoblotting. All tested IN mutant proteins were incorporated into MMTV particles at levels similar to the WT IN (Supplementary Figure S11B). IN mutant control viruses D122N and delIN supported ∼10% and 1% of the level of WT MMTV infectivity, respectively (Figure 5A, B). Whereas IN mutants P125D, Y149G and D223R supported similar levels of MMTV infection as IN mutant D122N, P125T and D223A were about twice as infectious, displaying ∼20% of WT MMTV infectivity.

We next determined sites of WT and IN mutant MMTV integration in human genomic DNA. For this, DNAs purified from infected cells were fragmented using restriction endonuclease digestion and amplified by ligation-mediated (LM)-PCR to enhance detection of virus U5 end-host sequences by Illumina sequencing. Raw reads filtered for *bona fide* MMTV-host junction sequences yielded between approximately 2300 and 18 500 unique integration sites for the different viruses, with the largest integration site dataset expectedly coming from cells infected with WT MMTV (Figure 5C). Integration frequencies relative to human genomic annotations such as RefSeq genes and gene-dense regions were compared to computer-generated random integration control (RIC) values that were based on the known frequencies of restriction enzyme sites in the human genome. As expected (26,73,74), WT MMTV disfavored integration into transcriptionally-active chromatin, as evidenced by lower frequencies of integration into RefSeq genes (*P* = 4.1 × 10^–8^) and gene-dense regions (*P* = 1.4 × 10^–10^) compared to matching RIC values (Figure 5C and Supplementary Table S3). Moreover, the frequency of WT MMTV integration in the vicinity of SPADs, a separate marker of active chromatin (75), was indistinguishable from random (*P* = 0.22). The integration site patterns of the different IN mutant viruses generally mimicked those of WT MMTV. While each mutant was comparatively enriched for integration in gene-dense regions versus the WT, the P125T virus was also enriched for integration into genes versus the WT (*P* = 0.003) and for integration in gene dense regions versus random (*P* = 0.002) (Figure 5C and Supplementary Table S3).

Target DNA sequences surrounding the virus–host junctions were aligned to examine frequencies of nucleotide usage during WT and IN mutant viral integration. DNA sequence logo analysis revealed periodic selection of A/T nucleotides emanating out from either side of the TSD, consistent with integration into nucleosomal DNA (27,76) (Figure 5D). In agreement with our previous analyses (26), and as evidenced by tDNA bending in the STC structure, flexible YR dinucleotides were enriched in the center of WT MMTV integration sites (Figure 5D). We also noted a marginal consensus ATN/GTTAACNAT sequence (the forward slash marks the point of vDNA U5 plus-strand joining to host DNA, and the underline denotes the 6-bp cleavage site) at sites of WT MMTV integration, including preference for G/C at positions 0/+5. Alignment of integration sites of MMTV IN mutant viruses revealed target site sequence preferences that were largely similar to those of WT MMTV (Supplementary Figure S11C). Although the absence of a change in phenotype among IN mutants Y149G and D223A/R was perhaps not surprising because these residues do not make base-specific contacts with tDNA, we anticipated that there would be some changes in local base selectivity for the P125T/D IN mutants based on prior results with similar substitutions in other retroviral IN proteins (8,25,27,30).

To further inform mutant viral integration phenotypes, we examined tDNA interactions with Pro125 analogous residues among retroviral STC structures. Because different tDNA sequences were used to assemble the complexes, we mutagenized *in silico* the +8 position of tDNA to an adenine, when appropriate, to afford consistency in these analyses. We then measured distances between the α-carbon of the sidechain at the position analogous to MMTV IN Pro125 and the N3 of the adenine base. These distances in the PFV STC (Supplementary Figure S12A), HIV-1 STC (Supplementary Figure S12B) and RSV STC (Supplementary Figure S12C) spanned from 4.1 to 4.8 Å, which would lead to steric clashes upon residue substitution and alter base stacking interactions that could affect tDNA base preferences at positions –3/+8, as directly observed for PFV (8) and HIV-1 (25,27). Similar effects can be indirectly inferred by changes in the *in vitro* integration patterns of analogous RSV IN mutant proteins (29). The structure of the MMTV STC provides a plausible explanation for why such changes were not observed for this virus. In contrast to the other INs, where Pro125-analogous residues situate comparatively close to tDNA bases, Pro125 positions marginally further from the tDNA (Supplementary Figure S12D). We speculate that this increased separation downplayed the alteration of tDNA base selection incurred via Pro125 mutations.

### B-to-A transition in tDNA is a general trait of retroviral integration

**Figure 6.**
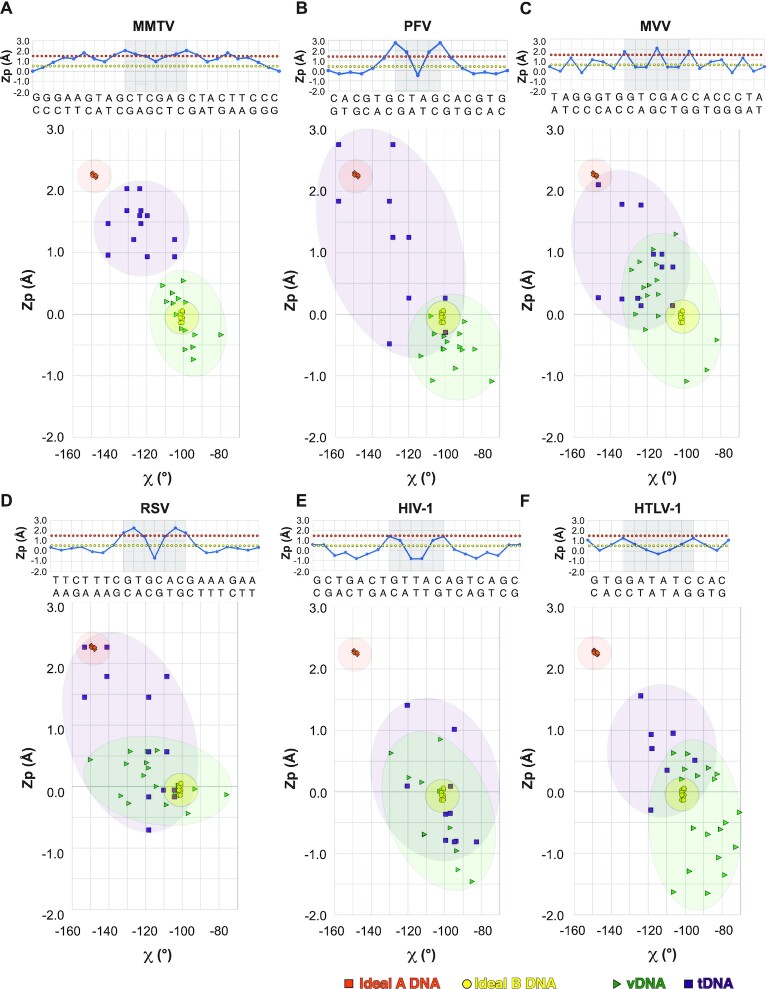
B- and A-DNA forms in retroviral STCs. Available retroviral STCs were analyzed for DNA conformation, including (**A**) MMTV (this work), (**B**) PFV (PDB ID: 3OS0), (**C**) MVV (PDB ID: 7Z1Z), (**D**) RSV (PDB ID: 5EJK), (**E**) HIV-1 (PDB ID: 5U1C) and (**F**) HTLV-1 (PDB ID: 6VOY). In each panel, the linear plots of Z_p_ for tDNA are displayed above, with cleavage sites denoted by light gray boxes, and *Z*_p_ versus χ are displayed below. For the *Z*_p_ versus χ plots, central cleavage sites ±4 bp are displayed, with the exception of the HTLV-1 STC, wherein the cleavage site ±2 bp is displayed due to limited resolution outside this region of the cryo-EM map. Values typical of A- versus B-DNA in the different panels are indicated by orange and yellow dotted lines (above) and shaded regions (below), respectively.

To determine whether A-form tDNA is a general feature of retroviral integration, we examined the available STC structures, specifically focusing on *Z*_p_ plots and *Z*_p_ versus χ plots, which can be used to distinguish DNA forms (71). Plots for the MMTV STC are displayed in Figure 6A for comparison. In the PFV STC, which is resolved to the highest resolution among the available structures, a clear transition to A-form DNA around the sites of vDNA joining was observed (Figure 6B). As evidenced from both the linear *Z*_p_ plots and *Z*_p_ versus χ plots, the analogous regions of tDNA within the MVV and RSV STCs also harbored A-like configurations (Figure 6C, D). This region of the HIV-1 STC tDNA trended toward the A-form in the *Z*_p_ plot, although the distinction was less clear in the *Z*_p_ versus χ plot (Figure 6E). The converse was true with HTLV-1 (Figure 6F). We note that both the HIV-1 and HTLV-I structures were resolved to lower resolution than the other STCs, and the tDNA in the HIV-1 structure was designed with a T/T mismatch in the center of the cleavage site. Resolution limits may affect the resulting models and interpretation of tDNA conformation, as was also observed for the PFV STC-nucleosome complex (see Materials and Methods), and the T/T mismatch affects bp stacking amid the HIV-1 tDNA cleavage sequence. However, in all cases, elevated *Z*_p_ values were observed around the sites of vDNA joining, and tDNA trended more toward A-form DNA than did the vDNAs.

Due to the constraints imposed by the above structure-based analysis, which limited tDNA assessment to the single sequence present within the respective STCs, we next analyzed sites of genomic DNA integration, where tens of thousands to millions of integration sites amass. A previous study of 100s of lentiviral, α- and γ-retroviral integration sites, which were determined by Sanger sequencing, indicated that retroviruses prefer to integrate into A-philic tDNA sites, but the comparatively low number of mapped sites limited the bp resolutions of these analyses (77). We have accordingly leveraged massively increased numbers of genomic integration sites afforded by next-generation sequencing technologies, which yielded from ∼20 000 to >4 million sites per dataset, to assess A-philicty profiles of retroviral integration sites.

**Figure 7.**
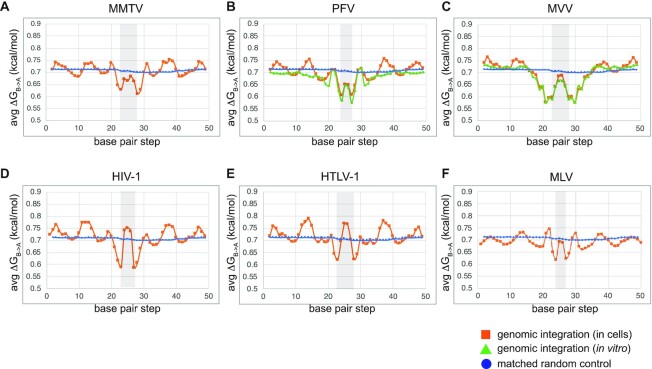
Free energy profiles associated with B-to-A transition in genomic DNA around retroviral integration sites. Average Δ*G*_B→A_ values were assigned to aligned trinucleotide positions within genomic integration sites and plotted against the bp step, tabulated based on values defined in Tolstorukov *et al.* (56). Plots are shown for genomic integration conducted either in cells (orange lines) or *in vitro*, wherein deproteinized genomic DNA was used as target (green lines). Plots refer to (**A**) MMTV (this work), (**B**) PFV *in vitro* (57) and in cells (58), (**C**) MVV *in vitro* (15) and in cells (19), (**D**) HIV-1 (52,59), (**E**) HTLV-1 (60) and (**F**) MLV (52).

In the absence of other variables, the propensity for A-philicity is sequence-dependent, facilitated by interactions between neighboring nucleotides (56). To quantify A-philicity at vDNA insertion sites, we adapted a free-energy based model for quantifying A-form DNA solely from sequence, based on experimental ΔG_B→A_ values for trinucleotides (56). We then tabulated mean Δ*G*_B→A_ values at each position in alignments of integration sites generated by infection of human cells with MMTV (this work), PFV (58), MVV (19), HIV-1 (52,59), HTLV-1 (60) and MLV (52). Integration sites of *in vitro* assembled PFV (57) and MVV (15) intasomes into deproteinized genomic DNA and *in silico*-generated RIC sequences were analyzed in parallel. The plots displaying mean Δ*G*_B→A_ values along alignments of integration sites for each dataset are shown in Figure 7A–F. Low Δ*G*_B→A_ values correspond to an increased propensity to form A-like DNA at a given position in tDNA, with consistent 0.7 to 0.72 values assessed for the RIC dataset. Two general trends were noted from the virus-specific patterns: (i) trinucleotides consistently displayed the greatest intrinsic propensity to form A-DNA around the sites of vDNA joining; (ii) A-philic peaks were separated by the spacing of vDNA joining, namely 6-bp for MMTV, MVV (where additional peaks were observed 2 bp outside the TSD), and HTLV-1, 5-bp for HIV-1 and 4-bp for PFV and MLV, strongly supporting the idea that A-form tDNA facilitates TCC intasome formation and IN strand transfer activity. In comparing cellular and *in vitro* PFV and MVV integration sites (Figure 7B, C), we noted highly similar profiles in and immediately adjacent to the TSDs, with notable divergence outside from these central regions. The periodic preferences for B-form DNA specific to integration in cells may reflect nucleosome occupancy, which is consistent with the known disfavoritism for A-form DNA (78). Collectively, these data support what was implied by the structural data, strongly arguing for the generality of B-to-A transition in tDNA during retroviral integration.

## DISCUSSION

### Target DNA bending and its role in retroviral integration

Intasome binding induces a severe bend in tDNA through extensive interactions between IN and the tDNA backbone, predominantly downstream of the cleavage site. Such interactions are observed within the MMTV STC, and, more generally, in other retroviral STCs (Supplementary Figure S9). To accommodate the bend, the target site must be flexible, which is visualized structurally (Figure 2A) (19) and inferred through dinucleotide step analysis (Figure 5C) (26). Retroviral STCs consistently display bent target sites, and sequences preferentially selected by retroviral INs are expected to be bendable (8,25–27,77). A simple analogy for the requirement of tDNA bending can be made to the action of a spring-loaded ratchet arm. In such a device, force is applied onto each of two extended lever arms connected by a central pivoting spring. Force loading builds up tension in and around the pivot point, storing potential energy that can, in turn, be converted into work. In this analogy, the deformable target site is the central spring-loaded pivot, and the interactions of IN with the tDNA backbone generate the force required to bend the tDNA. Collectively, the force applied onto the tDNA, the bendability of the tDNA at and around the cleavage sites, and the potential energy released through IN-catalyzed tDNA strand cleavage collectively facilitate the forward S_N_2 transesterification reaction (strand transfer). The observed deformations in rise and roll at the central tDNA dinucleotide further support this perspective (Figure 2D, E). Transcription factors are known to leverage amino acid side chains to intercalate DNA bases and elicit analogous deformations (79). However, in retroviral STCs, these deformations arise in response to distant contacts, consistent with the notion that force transferred from a distance plays a key role in tDNA bending and integration.

The evolutionary significance of tDNA bending and its recognition by retroviral intasomes can be rationalized through two important requirements for the catalysis of strand transfer. First, bent tDNA ensures proper spacing of metal cation-coordinated IN active sites to position optimally for concerted integration of two vDNA ends. Second, the constraints of the bent tDNA are released upon strand transfer, thereby suppressing the backward disintegration reaction via reconfiguration of the active site, as was experimentally observed for the PFV intasome (8,9) and for the more distantly related Mu phage transpososome (80).

### B-form to A-form transition in tDNA

A notable feature of the MMTV STC structure is the pronounced tDNA deformation in and around the sites of vDNA joining. Although minor groove compression and major groove widening were previously observed for retroviral STCs (8), the characteristics and implications of tDNA deformation were not fully appreciated. We now show that the tDNA specifically around the cleavage sites in the MMTV STC structure closely resembles the A-form, as evidenced by gradual increases in the Z_p_, and decreases in Slide, Twist, and χ (Figure 2B-C). We note that, consistent with the preference for G/C bp occupying A-DNA forms (56), target sites selected by retroviral INs consistently favor either a G or a C immediately following the cleavage site (Figure 5D and references (8,22–28,30)). Although pure A-form DNA is also characterized by a compressed major groove, which is not observed here, it is well-known that widened major grooves can be encountered immediately next to A-form DNAs (71); moreover, the features of DNA in high-resolution structures can vary when compared to the classical definitions based on canonical fiber-diffraction models (71).

There are many examples of B-to-A deformations among nucleoprotein complexes that cut and/or seal DNA at the O3′–P phosphodiester linkage (71). In support of our structural findings, prior computational studies suggested that regions in the vicinity of retroviral integration sites had elevated A-philicity scores (77). However, the previous analysis was limited by the comparatively low number of integration sites available at the time. Using massively expanded retroviral integration site data available today, we determined largely symmetrical peaks of A-philicity at sites of vDNA joining in genomic DNA (Figure 7). The low Δ*G*_B→A_ values associated with trinucleotide sequences selected by all examined retroviruses at the genomic level, together with our STC analyses (Figures 2 and 6), strongly argue for a general preference of A-form tDNA at sites of vDNA joining. Based on our data, we propose that retroviral integration is accompanied by a local transition of B-form tDNA to the A-form. Prior to IN binding and formation of the TCC intasome, we envision genomic tDNA is predominantly B-form. The propensity for certain genomic sequences to adopt A-form, as evident by A-philicity scores (Figure 7), favors TCC intasome formation and the juxtaposition of tDNA scissile phosphodiester bonds at the two IN active sites. Finally, strand transfer stabilizes A-form configurations at and around the sites of vDNA joining, as evidenced through structural analyses of diverse STC intasomes (Figures 2 and 6).

The B-to-A transition provides a mechanism for smoothly bending the double helix (81), which has broad implications for a variety of biological processes. Among nucleases, the main purpose of the transition is to selectively expose sugar-phosphate atoms for enzymatic cleavage, which are otherwise buried within the backbone chain in B-form DNA (82,83). It is further worth noting that, despite its prevalence within nucleoprotein complexes, A-form DNA is less favored under physiological conditions. In support of this idea, molecular dynamics simulations indicate that the A-form quickly transitions to the B-form once bond restraints are released (84). In the context of integration, A-form tDNA may thus be mechanistically important not only for strand transfer, but also for ensuing events. Once strand transfer is complete, the energy gained from transitioning from the A-form back to the B-form could facilitate target site melting and STC disassembly. However, unlike free DNA dynamics, this transition of A- to B-DNA in the context of STC disassembly is expected to be slow (85).

### The role of base-specific interactions in tDNA cleavage

Retroviral STC structures have revealed several instances of base-specific IN-tDNA contacts (8,10,12,17). Given the very low sequence preference at integration sites, the likely role for such interactions is to compensate for energetically unfavorable tDNA deformations. The most conserved interacting region is the α2 helix of the CCD, which includes Pro125 of MMTV IN and analogous small chain residues from other retroviral INs that stabilize sequences flanking the sites of vDNA joining (Supplementary Figure S10). A second interaction typically occurs through the β1-β2 loop of the CTD, including Arg231 (HIV-1 and MVV), Arg329 (PFV) or Glu229 (RSV), which stabilizes the deformed target site. However, a similar contact is missing in the MMTV STC. As highlighted in the results section (Supplementary Figure S10), two regions of IN-tDNA contacts, which encompass the IN CTD β1-β2 loop and MMTV IN Tyr149 analogues, vary across retroviral STCs. Stabilizing IN-tDNA interactions need to conform to the peculiarities of each intasome, which in turn account for the exact nature of the central tDNA bend, the distance between the two active sites, and more broadly the overall architecture of the complex and how it influences the tDNA path as a whole. The relative sparsity of base-specific tDNA interactions among retroviral intasomes distinguishes these complexes from certain transpososomes, including those from the IS630/Tc1/mariner family, which leverage tDNA sequence-specificity for selective transposition (4,86–89).

In PFV, HIV-1, RSV and MLV, substituting the tDNA binding residue in the α2 helix of the CCD with short side-chain amino acids alters integration sequence preferences (8,27,29,30). Long side-chain amino acid substitutions like glutamate are detrimental to viral fitness, presumably due to steric hindrance and/or charge repulsion affecting the tDNA interaction, and possibly the ability to optimally bend tDNA flanking the cleavage sites. Somewhat surprisingly, in MMTV, substitutions P125T and P125D did not yield gross changes in sequence logos at sites of vDNA integration (Supplementary Figure S11C). The preference for flexible A/T persisted 2–3 bp away from either side of the tDNA cleavage sites, irrespective of the residue tested. However, these substitutions did cause diminished infectivity and reduced IN concerted integration activity *in vitro*. Modeling threonine or aspartate side chains at position 125 suggests these substitutions would still make Van der Waals interactions with tDNA without introducing substantial steric clashes, and the effective distance of the side-chain to base would not change significantly in comparison to the distance measured for Pro125 (Supplementary Figure S12D). In fact, numerous *in silico* substitutions of MMTV IN Pro125 maintained side-chain–tDNA base distances that were greater than similar mutations among other retroviral INs, such as HIV-1, PFV and RSV. Thus, the architecture of the tDNA, especially around the cleavage sites, can permit different amino acid substitutions without compromising intasome binding, and by extension, tDNA bending.

Although P125T/D substitutions did not noticeably impact nucleobase selectivity at sites of MMTV integration, they did marginally impact the frequencies at which global genomic annotations, such as RefSeq genes and gene dense regions, were targeted (Figure 5C). Due to the sizes of the respective integration site datasets, the increase in P125D IN mutant gene targeting was less statistically robust compared to the difference between P125T and WT (respective *P*-values of 0.02 and 0.003; see Supplementary Table S3 for details). Nevertheless, these findings are consistent with the previous report that alterations of Ser119 in HIV-1 IN, which is analogous to Pro125 in MMTV IN, can impact global targeting frequencies of HIV-1 integration (25).

### An expanded target site marked by flexible dinucleotides

DNA distortions depend on the intrinsic structure and deformability of the base-pair sequence to which the protein is bound. INs that cleave DNA with 5- or 6-bp spacing, which include HIV-1, MMTV, RSV, HTLV-1 and MVV, prefer tDNA containing a more obtuse angle in the region immediately surrounding the cut sites, while PFV IN, which cleaves tDNA with 4-bp spacing, prefers tDNA that is bent substantially more sharply. Despite these differences, the tDNA at sites of vDNA joining is severely distorted in all STC and TCC structures. In addition, the flanking regions abutting the cleavage sites appear to be ubiquitously characterized by flexible dinucleotides (Figure 5C and (26)). Thus, tDNA flexibility is not just constrained to the region internal to the joined vDNA ends, but includes up to three flanking bp. This observation supports prior reports highlighting an expanded target site preferentially engaged by retroviral intasomes, which includes ±3–4 nt on either end of the cleavage site (26,27). The requirement to bend the expanded target site locally to promote forward integration puts further pressure on selectively stabilizing certain bp. Target DNA unwinding may further influence base stacking and selectivity in the regions flanking the target site and/or confer flexibility to the tDNA. Variations in the expanded target site may ultimately translate into differences in nucleosome engagement (90,91).

### Hyperactive half-site IN activity

We previously reported that R240E MMTV IN was defective for strand transfer activity *in vitro* and moreover inhibited the function of WT IN. The results supported the requirement for the flanking MMTV IN dimers in integration. However, Arg240 makes multiple interactions within the intasome assembly, thus warranting further analysis. Here, we mutated Asp223, which interacts with Arg240 via a salt bridge, to either alanine or arginine, and measured the activities of purified mutant proteins and viral infectivity. While we were unable to interpret D223A mutant IN 3′-processing activity due to apparent competing exonuclease activity derived from *E. coli*, D223R displayed about 30% of WT IN 3′-processing activity. Surprisingly, both D223A and D223R displayed robust h.s. integration activity alongside marginal c.i. activity. It is unclear from our data whether h.s. integration activity contributed to the infection phenotypes of D223A/R IN mutant viruses. Mutagenic or pharmacological enhancement of single end RSV (65,66) or HIV-1 (92) integration revealed that the second vDNA end is commonly integrated via host mechanisms, which can insert the rogue end distal from the IN-mediated event. In such cases, the tDNA consensus sequence would predictably differ from the WT ATN/GTTAACNAT, which was not observed (Supplementary Figure S11C). Apparently, residual levels of IN concerted integration activity primarily mediated the integration of D223A/R IN mutant viruses in cells.

To understand the structural basis of retroviral IN h.s. integration activity, we attempted to construct stable nucleoprotein complexes with D223A/R INs. These attempts, however, were unsuccessful. Thus, quasi-stable MMTV CSC intasomes may account for the observed hyper-stimulated h.s. integration activities of these mutant INs *in vitro*. In this scenario, the IN octamer would form, but then rapidly dissociate, on average leaving just a single vDNA engaged for IN catalysis. Perhaps more likely, due to disruption of the Asp223–Arg240 salt-bridge, these IN mutants may support the formation of substoichiometric IN-vDNA assemblies, resulting in the engagement of just a single vDNA, or two vDNA ends where one of the ends is mispositioned for integration into tDNA. In support of the latter hypothesis, different types of nucleoprotein complexes have been shown to mediate concerted versus half-site integration of HIV-1 DNA *in vitro*. Whereas SSCs/CSCs are obligate intermediates along the concerted integration pathway, quasi stable complexes promote h.s. integration (93,94). The pathway of retroviral intasome assembly is not well understood. Future studies aimed at assembling complexes that promote half-site versus concerted integration of different U3/U5 vDNAs should help to clarify this important area of research.

## DATA AVAILABILITY

The cryo-EM maps of the octameric MMTV STC and higher-order STC assembly were deposited into the EMDB under accession codes EMD-26737 and EMD-26744, respectively. Atomic models were deposited into the PDB under accession codes 7USF and 7UT1 for the two above maps, respectively. Illumina sequences derived from LM-PCR libraries of genomic DNA isolated from WT and IN mutant MMTV infections, deposited in the National Center for Biotechnology Information Sequence Read Archive (NCBI SRA), are accessible via PRJNA827605. The python script for analyzing free energy values associated with the B-to-A transition in genomic integration sites is available upon request.

## Supplementary Material

gkac644_Supplemental_Files

## Notes

Present address: Allison Ballandras-Colas, Institut de Biologie Structurale (IBS) CNRS, CEA, University Grenoble, Grenoble, France.

## References

[1] Fu S. , Phan A.T., Mao D., Wang X., Gao G., Goff S.P., Zhu Y. HIV-1 exploits the fanconi anemia pathway for viral DNA integration. Cell Rep. 2022; 39:110840. 35613597 10.1016/j.celrep.2022.110840 PMC9250337

[2] Maertens G.N. , Engelman A.N., Cherepanov P. Structure and function of retroviral integrase. Nat. Rev. Microbiol. 2022; 20:20–34. 34244677 10.1038/s41579-021-00586-9 PMC8671357

[3] Passos D.O. , Li M., Craigie R., Lyumkis D Retroviral integrase: structure, mechanism, and inhibition. The Enzymes. 2021; 50:Academic Press 249–300. 34861940 10.1016/bs.enz.2021.06.007 PMC8732146

[4] Nesmelova I.V. , Hackett P.B. DDE transposases: structural similarity and diversity. Adv. Drug Deliver. Rev. 2010; 62:1187–1195. 10.1016/j.addr.2010.06.006 PMC2991504 20615441

[5] Miller M.D. , Farnet C.M., Bushman F.D. Human immunodeficiency virus type 1 preintegration complexes: studies of organization and composition. J. Virol. 1997; 71:5382–5390. 9188609 10.1128/jvi.71.7.5382-5390.1997 PMC191777

[6] Lee M.S. , Craigie R. A previously unidentified host protein protects retroviral DNA from autointegration. Proc. Natl. Acad. Sci. U.S.A. 1998; 95:1528–1533. 9465049 10.1073/pnas.95.4.1528 PMC19075

[7] Hare S. , Gupta S.S., Valkov E., Engelman A., Cherepanov P. Retroviral intasome assembly and inhibition of DNA strand transfer. Nature. 2010; 464:232–236. 20118915 10.1038/nature08784 PMC2837123

[8] Maertens G.N. , Hare S., Cherepanov P. The mechanism of retroviral integration from X-ray structures of its key intermediates. Nature. 2010; 468:326–329. 21068843 10.1038/nature09517 PMC2999894

[9] Hare S. , Maertens G.N., Cherepanov P. 3’-processing and strand transfer catalysed by retroviral integrase in crystallo. EMBO J. 2012; 31:3020–3028. 22580823 10.1038/emboj.2012.118 PMC3395085

[10] Bhatt V. , Shi K., Salamango D.J., Moeller N.H., Pandey K.K., Bera S., Bohl T.E., Kurniawan F., Orellana K., Zhang W. et al. Structural basis of host protein hijacking in human T-cell leukemia virus integration. Nat. Commun. 2020; 11:3121. 32561747 10.1038/s41467-020-16963-6 PMC7305164

[11] Barski M.S. , Minnell J.J., Hodakova Z., Pye V.E., Nans A., Cherepanov P., Maertens G.N. Cryo-EM structure of the deltaretroviral intasome in complex with the PP2A regulatory subunit B56γ. Nat. Commun. 2020; 11:5043. 33028863 10.1038/s41467-020-18874-y PMC7542444

[12] Yin Z. , Shi K., Banerjee S., Pandey K.K., Bera S., Grandgenett D.P., Aihara H. Crystal structure of the Rous sarcoma virus intasome. Nature. 2016; 530:362–366. 26887497 10.1038/nature16950 PMC4881392

[13] Pandey K.K. , Bera S., Shi K., Rau M.J., Oleru A.V., Fitzpatrick J.A.J., Engelman A.N., Aihara H., Grandgenett D.P. Cryo-EM structure of the Rous sarcoma virus octameric cleaved synaptic complex intasome. Commun. Biol. 2021; 4:330. 33712691 10.1038/s42003-021-01855-2 PMC7955051

[14] Ballandras-Colas A. , Brown M., Cook N.J., Dewdney T.G., Demeler B., Cherepanov P., Lyumkis D., Engelman A.N. Cryo-EM reveals a novel octameric integrase structure for betaretroviral intasome function. Nature. 2016; 530:358–361. 26887496 10.1038/nature16955 PMC4908968

[15] Ballandras-Colas A. , Maskell D.P., Serrao E., Locke J., Swuec P., Jonsson S.R., Kotecha A., Cook N.J., Pye V.E., Taylor I.A. et al. A supramolecular assembly mediates lentiviral DNA integration. Science. 2017; 355:93–95. 28059770 10.1126/science.aah7002 PMC5321526

[16] Cook N.J. , Li W., Berta D., Badaoui M., Ballandras-Colas A., Nans A., Kotecha A., Rosta E., Engelman A.N., Cherepanov P. Structural basis of second-generation HIV integrase inhibitor action and viral resistance. Science. 2020; 367:806–810. 32001525 10.1126/science.aay4919 PMC7023979

[17] Passos D.O. , Li M., Yang R., Rebensburg S.V., Ghirlando R., Jeon Y., Shkriabai N., Kvaratskhelia M., Craigie R., Lyumkis D Cryo-EM structures and atomic model of the HIV-1 strand transfer complex intasome. Science. 2017; 355:89–92. 28059769 10.1126/science.aah5163 PMC5508583

[18] Passos D.O. , Li M., Jóźwik I.K., Zhao X.Z., Santos-Martins D., Yang R., Smith S.J., Jeon Y., Forli S., Hughes S.H. et al. Structural basis for strand-transfer inhibitor binding to HIV intasomes. Science. 2020; 367:810–814. 32001521 10.1126/science.aay8015 PMC7357238

[19] Ballandras-Colas A. , Chivukula V., Gruszka D.T., Shan Z., Singh P.K., Pye V.E., McLean R.K., Bedwell G.J., Li W., Nans A. et al. Multivalent interactions essential for lentiviral integrase function. Nat. Commun. 2022; 13:2416. 35504909 10.1038/s41467-022-29928-8 PMC9065133

[20] Engelman A.N. , Cherepanov P. Retroviral intasomes arising. Curr. Opin. Struct. Biol. 2017; 47:23–29. 28458055 10.1016/j.sbi.2017.04.005 PMC5660667

[21] Engelman A.N. , Maertens G.N. Virus-host interactions in retrovirus integration. Retrovirus-Cell interactions. 2018; Academic Press 163–198.

[22] Holman A.G. , Coffin J.M. Symmetrical base preferences surrounding HIV-1, avian sarcoma/leukosis virus, and murine leukemia virus integration sites. Proc. Natl. Acad. Sci. U.S.A. 2005; 102:6103–6107. 15802467 10.1073/pnas.0501646102 PMC1087937

[23] Stevens S.W. , Griffith J.D. Sequence analysis of the human DNA flanking sites of human immunodeficiency virus type 1 integration. J. Virol. 1996; 70:6459–6462. 8709282 10.1128/jvi.70.9.6459-6462.1996 PMC190680

[24] Derse D. , Crise B., Li Y., Princler G., Lum N., Stewart C., McGrath C.F., Hughes S.H., Munroe D.J., Wu X. Human T-cell leukemia virus type 1 integration target sites in the human genome: comparison with those of other retroviruses. J. Virol. 2007; 81:6731–6741. 17409138 10.1128/JVI.02752-06 PMC1900082

[25] Demeulemeester J. , Vets S., Schrijvers R., Madlala P., Maeyer M.D., Rijck J.D., Ndung’u T., Debyser Z., Gijsbers R. HIV-1 integrase variants retarget viral integration and are associated with disease progression in a chronic infection cohort. Cell Host Microbe. 2014; 16:651–662. 25525795 10.1016/j.chom.2014.09.016

[26] Serrao E. , Ballandras-Colas A., Cherepanov P., Maertens G.N., Engelman A.N. Key determinants of target DNA recognition by retroviral intasomes. Retrovirology. 2015; 12:39. 25924943 10.1186/s12977-015-0167-3 PMC4422553

[27] Serrao E. , Krishnan L., Shun M.-C., Li X., Cherepanov P., Engelman A., Maertens G.N. Integrase residues that determine nucleotide preferences at sites of HIV-1 integration: implications for the mechanism of target DNA binding. Nucleic Acids Res. 2014; 42:5164–5176. 24520116 10.1093/nar/gku136 PMC4005685

[28] Kirk P.D.W. , Huvet M., Melamed A., Maertens G.N., Bangham C.R.M. Retroviruses integrate into a shared, non-palindromic DNA motif. Nat. Microbiol. 2016; 2:16212. 27841853 10.1038/nmicrobiol.2016.212 PMC7613964

[29] Harper A.L. , Sudol M., Katzman M. An amino acid in the central catalytic domain of three retroviral integrases that affects target site selection in nonviral DNA. J. Virol. 2003; 77:3838–3845. 12610159 10.1128/JVI.77.6.3838-3845.2003 PMC149511

[30] Aiyer S. , Rossi P., Malani N., Schneider W.M., Chandar A., Bushman F.D., Montelione G.T., Roth M.J. Structural and sequencing analysis of local target DNA recognition by MLV integrase. Nucleic Acids Res. 2015; 43:5647–5663. 25969444 10.1093/nar/gkv410 PMC4477651

[31] Nusse R. , Varmus H.E. Many tumors induced by the mouse mammary tumor virus contain a provirus integrated in the same region of the host genome. Cell. 1982; 31:99–109. 6297757 10.1016/0092-8674(82)90409-3

[32] Goubran M. , Wang W., Indik S., Faschinger A., Wasilenko S.T., Bintner J., Carpenter E.J., Zhang G., Nuin P., Macintyre G. et al. Isolation of a human betaretrovirus from patients with primary biliary cholangitis. Viruses. 2022; 14:886. 35632628 10.3390/v14050886 PMC9146342

[33] Xu L. , Shen Z., Guo L., Fodera B., Keogh A., Joplin R., O’Donnell B., Aitken J., Carman W., Neuberger J. et al. Does a betaretrovirus infection trigger primary biliary cirrhosis. Proc. Natl. Acad. Sci. U.S.A. 2003; 100:8454–8459. 12832623 10.1073/pnas.1433063100 PMC166250

[34] Ballandras-Colas A. , Naraharisetty H., Li X., Serrao E., Engelman A. Biochemical characterization of novel retroviral integrase proteins. PLoS One. 2013; 8:e76638. 24124581 10.1371/journal.pone.0076638 PMC3790719

[35] Konstantoulas C.J. , Indik S. Mouse mammary tumor virus-based vector transduces non-dividing cells, enters the nucleus via a TNPO3-independent pathway and integrates in a less biased fashion than other retroviruses. Retrovirology. 2014; 11:34. 24779422 10.1186/1742-4690-11-34 PMC4098793

[36] Dull T. , Zufferey R., Kelly M., Mandel R.J., Nguyen M., Trono D., Naldini L. A third-generation lentivirus vector with a conditional packaging system. J. Virol. 1998; 72:8463–8471. 9765382 10.1128/jvi.72.11.8463-8471.1998 PMC110254

[37] Shun M.-C. , Daigle J.E., Vandegraaff N., Engelman A. Wild-Type levels of human immunodeficiency virus type 1 infectivity in the absence of cellular emerin protein. J. Virol. 2007; 81:166–172. 17035312 10.1128/JVI.01953-06 PMC1797258

[38] Punjani A. , Rubinstein J.L., Fleet D.J., Brubaker M.A. cryoSPARC: algorithms for rapid unsupervised cryo-EM structure determination. Nat. Methods. 2017; 14:290–296. 28165473 10.1038/nmeth.4169

[39] Tan Y.Z. , Baldwin P.R., Davis J.H., Williamson J.R., Potter C.S., Carragher B., Lyumkis D Addressing preferred specimen orientation in single-particle cryo-EM through tilting. Nat. Methods. 2017; 14:793–796. 28671674 10.1038/nmeth.4347 PMC5533649

[40] Sanchez-Garcia R. , Gomez-Blanco J., Cuervo A., Carazo J.M., Sorzano C.O.S., Vargas J. DeepEMhancer: a deep learning solution for cryo-EM volume post-processing. Commun. Biol. 2021; 4:874. 34267316 10.1038/s42003-021-02399-1 PMC8282847

[41] Hart D. , Cianfrocco M.A., Wong-Barnum M., Youn C., Wagner R., Leschziner A. COSMIC2. Proc. Pract. Exp. Adv. Res. Comput. 2017 Sustain Success Impact. 2017; 10.1145/3093338.3093390.

[42] Pettersen E.F. , Goddard T.D., Huang C.C., Couch G.S., Greenblatt D.M., Meng E.C., Ferrin T.E. UCSF Chimera–a visualization system for exploratory research and analysis. J. Comput. Chem. 2004; 25:1605–1612. 15264254 10.1002/jcc.20084

[43] Emsley P. , Lohkamp B., Scott W.G., Cowtan K. Features and development of Coot. Acta Crystallogr. D Biol. Crystallogr. 2010; 66:486–501. 20383002 10.1107/S0907444910007493 PMC2852313

[44] Adams P.D. , Afonine P.V., Bunkóczi G., Chen V.B., Davis I.W., Echols N., Headd J.J., Hung L.-W., Kapral G.J., Grosse-Kunstleve R.W. et al. PHENIX: a comprehensive Python-based system for macromolecular structure solution. Acta Crystallogr. D Biol. Crystallogr. 2010; 66:213–221. 20124702 10.1107/S0907444909052925 PMC2815670

[45] Afonine P.V. , Klaholz B.P., Moriarty N.W., Poon B.K., Sobolev O.V., Terwilliger T.C., Adams P.D., Urzhumtsev A. New tools for the analysis and validation of cryo-EM maps and atomic models. Acta Crystallogr. D Struct. Biol. 2018; 74:814–840. 30198894 10.1107/S2059798318009324 PMC6130467

[46] Williams C.J. , Headd J.J., Moriarty N.W., Prisant M.G., Videau L.L., Deis L.N., Verma V., Keedy D.A., Hintze B.J., Chen V.B. et al. MolProbity: more and better reference data for improved all-atom structure validation. Protein Sci. 2018; 27:293–315. 29067766 10.1002/pro.3330 PMC5734394

[47] Robert X. , Gouet P. Deciphering key features in protein structures with the new ENDscript server. Nucleic Acids Res. 2014; 42:W320–W324. 24753421 10.1093/nar/gku316 PMC4086106

[48] Sagendorf J.M. , Berman H.M., Rohs R. DNAproDB: an interactive tool for structural analysis of DNA–protein complexes. Nucleic Acids Res. 2017; 45:W89–W97. 28431131 10.1093/nar/gkx272 PMC5570235

[49] Sagendorf J.M. , Markarian N., Berman H.M., Rohs R. DNAproDB: an expanded database and web-based tool for structural analysis of DNA–protein complexes. Nucleic Acids Res. 2020; 48:D277–D287. 31612957 10.1093/nar/gkz889 PMC7145614

[50] Lu X. , Olson W.K. 3DNA: a software package for the analysis, rebuilding and visualization of three-dimensional nucleic acid structures. Nucleic Acids Res. 2003; 31:5108–5121. 12930962 10.1093/nar/gkg680 PMC212791

[51] Wilson M.D. , Renault L., Maskell D.P., Ghoneim M., Pye V.E., Nans A., Rueda D.S., Cherepanov P., Costa A. Retroviral integration into nucleosomes through DNA looping and sliding along the histone octamer. Nat. Commun. 2019; 10:4189. 31519882 10.1038/s41467-019-12007-w PMC6744463

[52] Li W. , Singh P.K., Sowd G.A., Bedwell G.J., Jang S., Achuthan V., Oleru A.V., Wong D., Fadel H.J., Lee K. et al. CPSF6-dependent targeting of speckle-associated domains distinguishes primate from nonprimate lentiviral integration. Mbio. 2020; 11:e02254-20. 32994325 10.1128/mBio.02254-20 PMC7527728

[53] Serrao E. , Cherepanov P., Engelman A.N. Amplification, next-generation sequencing, and genomic DNA mapping of retroviral integration sites. J. Vis. Exp. 2016; 53840. 10.3791/53840 PMC4829050 27023428

[54] Francis A.C. , Marin M., Singh P.K., Achuthan V., Prellberg M.J., Palermino-Rowland K., Lan S., Tedbury P.R., Sarafianos S.G., Engelman A.N. et al. HIV-1 replication complexes accumulate in nuclear speckles and integrate into speckle-associated genomic domains. Nat. Commun. 2020; 11:3505. 32665593 10.1038/s41467-020-17256-8 PMC7360574

[55] Crooks G.E. , Hon G., Chandonia J.-M., Brenner S.E. WebLogo: a sequence logo generator. Genome Res. 2004; 14:1188–1190. 15173120 10.1101/gr.849004 PMC419797

[56] Tolstorukov M.Y. , Ivanov V.I., Malenkov G.G., Jernigan R.L., Zhurkin V.B. Sequence-dependent B↔A transition in DNA evaluated with dimeric and trimeric scales. Biophys. J. 2001; 81:3409–3421. 11721003 10.1016/S0006-3495(01)75973-5 PMC1301797

[57] Maskell D.P. , Renault L., Serrao E., Lesbats P., Matadeen R., Hare S., Lindemann D., Engelman A.N., Costa A., Cherepanov P. Structural basis for retroviral integration into nucleosomes. Nature. 2015; 523:366–369. 26061770 10.1038/nature14495 PMC4530500

[58] Lesbats P. , Serrao E., Maskell D.P., Pye V.E., O’Reilly N., Lindemann D., Engelman A.N., Cherepanov P Structural basis for spumavirus GAG tethering to chromatin. Proc. Natl. Acad. Sci. U.S.A. 2017; 114:5509–5514. 28490494 10.1073/pnas.1621159114 PMC5448199

[59] Sowd G.A. , Serrao E., Wang H., Wang W., Fadel H.J., Poeschla E.M., Engelman A.N. A critical role for alternative polyadenylation factor CPSF6 in targeting HIV-1 integration to transcriptionally active chromatin. Proc. Nat. Acad. Sci. U.S.A. 2016; 113:E1054–E1063. 10.1073/pnas.1524213113 PMC4776470 26858452

[60] Melamed A. , Fitzgerald T.W., Wang Y., Ma J., Birney E., Bangham C.R.M. Selective clonal persistence of human retroviruses in vivo: radial chromatin organization, integration site, and host transcription. Sci. Adv. 2022; 8:eabm6210. 35486737 10.1126/sciadv.abm6210 PMC9054021

[61] Quinlan A.R. , Hall I.M. BEDTools: a flexible suite of utilities for comparing genomic features. Bioinformatics. 2010; 26:841–842. 20110278 10.1093/bioinformatics/btq033 PMC2832824

[62] Mitchell R.S. , Beitzel B.F., Schroder A.R.W., Shinn P., Chen H., Berry C.C., Ecker J.R., Bushman F.D. Retroviral DNA integration: ASLV, HIV, and MLV show distinct target site preferences. PLoS Biol. 2004; 2:e234. 15314653 10.1371/journal.pbio.0020234 PMC509299

[63] Narezkina A. , Taganov K.D., Litwin S., Stoyanova R., Hayashi J., Seeger C., Skalka A.M., Katz R.A. Genome-wide analyses of avian sarcoma virus integration sites. J. Virol. 2004; 78:11656–11663. 15479807 10.1128/JVI.78.21.11656-11663.2004 PMC523270

[64] Barr S.D. , Leipzig J., Shinn P., Ecker J.R., Bushman F.D. Integration targeting by avian sarcoma-leukosis virus and human immunodeficiency virus in the chicken genome. J. Virol. 2005; 79:12035–12044. 16140779 10.1128/JVI.79.18.12035-12044.2005 PMC1212630

[65] Oh J. , Chang K.W., Alvord W.G., Hughes S.H. Alternate polypurine tracts affect Rous sarcoma virus integration in vivo. J. Virol. 2006; 80:10281–10284. 17005708 10.1128/JVI.00361-06 PMC1617299

[66] Oh J. , Chang K.W., Hughes S.H. Mutations in the U5 sequences adjacent to the primer binding site do not affect tRNA cleavage by Rous sarcoma virus RNase H but do cause aberrant integrations in vivo. J. Virol. 2006; 80:451–459. 16352569 10.1128/JVI.80.1.451-459.2006 PMC1317513

[67] Yin Z. , Lapkouski M., Yang W., Craigie R. Assembly of prototype foamy virus strand transfer complexes on product DNA bypassing catalysis of integration. Protein Sci. 2012; 21:1849–1857. 23011895 10.1002/pro.2166 PMC3575915

[68] Majors J.E. , Varmus H.E. Nucleotide sequences at host–proviral junctions for mouse mammary tumour virus. Nature. 1981; 289:253–258. 6256658 10.1038/289253a0

[69] Ellison V. , Brown P.O. A stable complex between integrase and viral DNA ends mediates human immunodeficiency virus integration in vitro. Proc. Natl. Acad. Sci. U.S.A. 1994; 91:7316–7320. 8041787 10.1073/pnas.91.15.7316 PMC44390

[70] Russo C.J. , Passmore L.A. Electron microscopy: ultrastable gold substrates for electron cryomicroscopy. Science. 2014; 346:1377–1380. 25504723 10.1126/science.1259530 PMC4296556

[71] Lu X.-J. , Shakked Z., Olson W.K. A-form conformational motifs in ligand-bound DNA structures. J. Mol. Biol. 2000; 300:819–840. 10891271 10.1006/jmbi.2000.3690

[72] Choy G. , OConnor S., Diehn F.E., Costouros N., Alexander H.R., Choyke P., Libutti S.K. Comparison of noninvasive fluorescent and bioluminescent small animal optical imaging. BioTechniques. 2003; 35:1022–1030. 14628676 10.2144/03355rr02

[73] de Jong J. , Akhtar W., Badhai J., Rust A.G., Rad R., Hilkens J., Berns A., Lohuizen M.van, Wessels L.F.A., de Ridder J. Chromatin landscapes of retroviral and transposon integration profiles. PLoS Genet. 2014; 10:e1004250. 24721906 10.1371/journal.pgen.1004250 PMC3983033

[74] Faschinger A. , Rouault F., Sollner J., Lukas A., Salmons B., Günzburg W.H., Indik S. Mouse mammary tumor virus integration site selection in human and mouse genomes. J. Virol. 2008; 82:1360–1367. 18032509 10.1128/JVI.02098-07 PMC2224419

[75] Chen Y. , Zhang Y., Wang Y., Zhang L., Brinkman E.K., Adam S.A., Goldman R., Steensel B.van, Ma J., Belmont A.S. Mapping 3D genome organization relative to nuclear compartments using TSA-Seq as a cytological ruler. J. Cell Biol. 2018; 217:4025–4048. 30154186 10.1083/jcb.201807108 PMC6219710

[76] Wang G.P. , Ciuffi A., Leipzig J., Berry C.C., Bushman F.D. HIV integration site selection: analysis by massively parallel pyrosequencing reveals association with epigenetic modifications. Genome Res. 2007; 17:1186–1194. 17545577 10.1101/gr.6286907 PMC1933515

[77] Wu X. , Li Y., Crise B., Burgess S.M., Munroe D.J. Weak palindromic consensus sequences are a common feature found at the integration target sites of many retroviruses. J. Virol. 2005; 79:5211–5214. 15795304 10.1128/JVI.79.8.5211-5214.2005 PMC1069554

[78] Hovatter K.R. , Martinson H.G. Ribonucleotide-induced helical alteration in DNA prevents nucleosome formation. Proc. Natl. Acad. Sci. U.S.A. 1987; 84:1162–1166. 3493489 10.1073/pnas.84.5.1162 PMC304386

[79] Werner M.H. , Gronenborn A.M., Clore G.M. Intercalation, DNA kinking, and the control of transcription. Science. 1996; 271:778–784. 8628992 10.1126/science.271.5250.778

[80] Fuller J.R. , Rice P.A. Target DNA bending by the Mu transpososome promotes careful transposition and prevents its reversal. Elife. 2017; 6:e21777. 28177285 10.7554/eLife.21777 PMC5357137

[81] Selsing E. , Wells R.D., Alden C.J., Arnott S. Bent DNA: visualization of a base-paired and stacked A-B conformational junction. J. Biol. Chem. 1979; 254:5417–5422. 447660

[82] Horton N.C. , Perona J.J. Recognition of flanking DNA sequences by EcoRV endonuclease involves alternative patterns of water-mediated contacts. J. Biol. Chem. 1998; 273:21721–21729. 9705308 10.1074/jbc.273.34.21721

[83] Winkler F.K. , Banner D.W., Oefner C., Tsernoglou D., Brown R.S., Heathman S.P., Bryan R.K., Martin P.D., Petratos K., Wilson K.S. The crystal structure of EcoRV endonuclease and of its complexes with cognate and non-cognate DNA fragments. EMBO J. 1993; 12:1781–1795. 8491171 10.2210/pdb4rve/pdb PMC413397

[84] Waters J.T. , Lu X.-J., Galindo-Murillo R., Gumbart J.C., Kim H.D., Cheatham T.E., Harvey S.C. Transitions of double-stranded DNA between the A- and B-forms. J. Phys. Chem. B. 2016; 120:8449–8456. 27135262 10.1021/acs.jpcb.6b02155 PMC5267635

[85] Vanderlinden W. , Brouns T., Walker P.U., Kolbeck P.J., Milles L.F., Ott W., Nickels P.C., Debyser Z., Lipfert J. The free energy landscape of retroviral integration. Nat. Commun. 2019; 10:4738. 31628321 10.1038/s41467-019-12649-w PMC6802197

[86] Chen Q. , Luo W., Veach R.A., Hickman A.B., Wilson M.H., Dyda F. Structural basis of seamless excision and specific targeting by piggyBac transposase. Nat. Commun. 2020; 11:3446. 32651359 10.1038/s41467-020-17128-1 PMC7351741

[87] Ghanim G.E. , Kellogg E.H., Nogales E., Rio D.C. Structure of a P element transposase–DNA complex reveals unusual DNA structures and GTP-DNA contacts. Nat. Struct. Mol. Biol. 2019; 26:1013–1022. 31659330 10.1038/s41594-019-0319-6 PMC6948148

[88] Richardson J.M. , Colloms S.D., Finnegan D.J., Walkinshaw M.D. Molecular architecture of the Mos1 paired-end complex: the structural basis of DNA transposition in a eukaryote. Cell. 2009; 138:1096–1108. 19766564 10.1016/j.cell.2009.07.012 PMC3977044

[89] Miskey C. , Kesselring L., Querques I., Abrusán G., Barabas O., Ivics Z. Engineered sleeping beauty transposase redirects transposon integration away from genes. Nucleic Acids Res. 2022; 50:2807–2825. 35188569 10.1093/nar/gkac092 PMC8934666

[90] Taganov K.D. , Cuesta I., Daniel R., Cirillo L.A., Katz R.A., Zaret K.S., Skalka A.M. Integrase-specific enhancement and suppression of retroviral DNA integration by compacted chromatin structure in vitro. J. Virol. 2004; 78:5848–5855. 15140982 10.1128/JVI.78.11.5848-5855.2004 PMC415796

[91] Benleulmi M.S. , Matysiak J., Henriquez D.R., Vaillant C., Lesbats P., Calmels C., Naughtin M., Leon O., Skalka A.M., Ruff M. et al. Intasome architecture and chromatin density modulate retroviral integration into nucleosome. Retrovirology. 2015; 12:13. 25807893 10.1186/s12977-015-0145-9 PMC4358916

[92] Varadarajan J. , McWilliams M.J., Hughes S.H. Treatment with suboptimal doses of raltegravir leads to aberrant HIV-1 integrations. Proc. Nat. Acad. Sci. U.S.A. 2013; 110:14747–14752. 10.1073/pnas.1305066110 PMC3767498 23959861

[93] Li M. , Mizuuchi M., Burke T.R., Craigie R. Retroviral DNA integration: reaction pathway and critical intermediates. EMBO J. 2006; 25:1295–1304. 16482214 10.1038/sj.emboj.7601005 PMC1422164

[94] Pandey K.K. , Bera S., Zahm J., Vora A., Stillmock K., Hazuda D., Grandgenett D.P. Inhibition of human immunodeficiency virus type 1 concerted integration by strand transfer inhibitors which recognize a transient structural intermediate. J. Virol. 2007; 81:12189–12199. 17804497 10.1128/JVI.02863-06 PMC2169005

