# Supplementary material for: B-to-A transition in target DNA during retroviral integration
Source: Nucleic Acids Res. 2022 Aug 10;50(15):8898–918. doi: 10.1093/nar/gkac644 (PMC9410886; doi:10.1093/nar/gkac644)

Supplementary Data

B-to-A transition in target DNA during retroviral integration

Ilona K. Jóźwik, Wen Li, Da-Wei Zhang, Doris Wong, Julia Grawenhoff, Allison Ballandras-Colas, Sriram Aiyer, Peter Cherepanov, Alan N. Engelman, Dmitry Lyumkis

Supplementary Figures

Supplementary Tables

Supplementary References

Supplementary Figure Legends

Supplementary Figure S1. STC intasome assembly and purification. (**A**) STC intasome assembly pathways. The forward pathway is driven by IN-mediated catalysis of integration. The reverse assembly pathway uses bDNA that mimics the product of concerted integration. In both panels, vDNA is depicted in orange and tDNA is in black. (**B**) DNA sequence of the bDNA substrate used for STC intasome assembly. Phylogenetically-conserved CA dinucleotides are underlined. (**C**) Gel filtration profile of the STC intasome assembly reaction. Column fractions corresponding to the material within the black rectangle were pooled, concentrated, and used for cryo-EM grid preparation.

Supplementary Figure S2. Cryo-EM data processing workflow. All data processing steps were performed in cryoSPARC. Detailed step-by-step description is provided in the Materials and Methods section. Processing produced two distinct maps: one corresponding to (primarily) a single STC (bottom left), and one corresponding to two STCs (bottom right). Scale bar for the micrograph is 200 Å.

Supplementary Figure S3. Analysis and validation of the primary MMTV STC map. Side (**A**) and top (**B**) views, colored by local resolution, estimated in cryoSPARC. **(C)** Distribution of projection orientations. (**D**) Surface sampling plot of the Fourier sampling, with the sampling compensation factor (SCF) value indicated. (**E**) Fourier shell correlation (FSC) curves for half-map and map-to-model, with FSC cutoffs 0.143 and 0.5 indicated. (**F**) Global resolution FSC curve overlaid onto a histogram of directional resolution value. Green lines refer to +/- 1 s.t.d. of directional resolution values. (**G**) 3DFSC shown as an isosurface for the least favorable cross-sectional view and colored by spatial frequency.

Supplementary Figure S4. Comparison of the MMTV STC and CSC intasome structures. (**A**) Side and (**B**) top views of the MMTV STC (orange) and the CSC (PDB ID: 3JCA, blue), overlaid on one another. The root mean square deviation between the aligned protein residues is 0.75 Å.

Supplementary Figure S5. Analysis and validation of the second map containing stacked STCs. Side (**A**) and top (**B**) views, colored by local resolution, estimated in cryoSPARC. Numbers in parentheses denote two different STCs. **(C)** Distribution of projection orientations. (**D**) Surface sampling plot of the Fourier sampling, with the SCF value indicated. (**E**) FSC curves for half-map and map-to-model, with FSC cutoffs 0.143 and 0.5 indicated. (**F**) Global resolution FSC curve overlaid onto a histogram of directional resolution value. Green lines refer to +/- 1 s.t.d. of directional resolution values. (**G**) 3DFSC shown as an isosurface for the least favorable cross-sectional view and colored by spatial frequency.

Supplementary Figure S6. Structural basis of higher-order MMTV STC intasome formation. (**A-B**) A Gaussian filtered map of the STC revealed additional density that enabled rigid-body docking of two

additional STC intasomes, providing a composite model of 3 STCs stacked together. (**C-D**) The 3.8 Å map and the refined atomic model of two stacked MMTV STC intasomes. (**E-G**) Close-up of three inter-STC interfaces from the model. (**E**) Interaction between the tDNA of one STC and the CCD of another. (**F**) Flanking CCD-core CCD interactions between STCs. (**G**) Inter-STC flanking CCD-flanking CCD interactions. IN residues within 5 Å of each domain are shown in panels F and G.

Supplementary Figure S7. Base-specific interactions between IN and nucleic acids within the MMTV STC. Schematic shows the interactions between IN protomers and nucleobases. Additionally, sidechains that insert into either the major or minor groove are displayed. Note the few tDNA interactions in comparison to vDNA interactions. Protein residues are labeled with one letter codes, respective residue number, and the protein chain identifier (*e.g.*, chain A for P125A). Target DNA bases are numbered by positions in the atomic model; C21/G26 bp at sites of vDNA joining correspond to C0/G5 numbering in the main text and figures. W.C. BP – Watson–Crick bp; Hoog. BP – Hoogsteen bp; Other BP – other bp. Analyses were performed using DNAproDB (48, 49).

Supplementary Figure S8. Backbone interactions between IN and nucleic acids within the MMTV STC. Schematic shows all interactions between IN protomers and the nucleic acid backbone. For tDNA, the predominant interactions are observed downstream from the sites of vDNA joining. Protein residues are labeled with one letter codes, respective residue number, and the protein chain identifier (*e.g.*, chain A for P125A). Target bp numbering is the same as in Supplementary Figure S7. W.C. BP - Watson–Crick bp; Hoog. BP – Hoogsteen bp; Other BP – other bp. Analyses were performed using DNAproDB (48, 49).

Supplementary Figure S9. Comparison of interactions between IN and tDNA among retroviral STCs. Six different STCs are compared, including MMTV (this work), PFV (PDB ID: 3OS0), HIV-1 (PDB ID: 5U1C), MVV (PDB ID: 7Z1Z), HTLV-1 (PDB ID: 6VOY) and RSV (PDB ID: 5EJK). For each case, backbone-specific interactions are shown at left, and base interactions or sidechain insertions into the tDNA grooves are at right. Each respective tDNA cleavage site is in gray (6-bp for MMTV, RSV, HTLV-1, and MVV; 5-bp for HIV-1; 4-bp for PFV), and the sites of vDNA joining, apparent as breaks in the phosphate backbone, are highlighted by horizontal red arrows. Other abbreviations are as in Supplementary Figures S7 and S8.

Supplementary Figure S10: Comparison of tDNA binding residues among retroviral STCs. (A) Close-up overlay of 6 STCs and their base-specific interactions. The zoomed representation is of the CTD β1-β2 loop and CCD α2 helix region. Sidechains of residues involved in base-specific interactions from the CTD include Arg329 from PFV (PDB ID: 3OS0, green), Arg231 from HIV-1 (PDB ID: 5U1C, cyan), Arg231 from MVV (PDB ID: 7Z1Z, magenta), and Glu229 from RSV (PDB ID: 5EJK, salmon pink). As HTLV-1 (white) and MMTV (yellow) INs lack corresponding interactions, amino acids from these proteins are omitted. Sidechains of residues involved in base-specific interactions from the CCD, including Ala188 from PFV,

Ser119 from HIV-1, Pro121 from MVV, Ser124 from RSV, Pro123 from HTLV-1, and Pro125 from MMTV, are also displayed (α2 helix region). Color scheme is the same as for the CTDs. Target DNA is represented in orange, except the respective cleavage sites, which are displayed in red. (**B**) Same as panel A, except only the interactions for HTLV-1 and MMTV are displayed. In addition, residues in the CTD β1-β2 loop that appear to stabilize Tyr147 (HTLV-1) and Tyr149 (MMTV) contacts with the tDNA backbone are displayed. (**C**) Same as in panel A, except only PFV, HIV-1, MVV, and RSV are displayed. (**D**) Structure-based alignment of IN CTD β1-β2 sequences. Chemically conserved and invariant residues across the alignment are shown in red type and white type with red highlight, respectively. Residues within MVV, RSV, PFV, and RSV β1-β2 loop regions that interact with tDNA bases are boxed in green. Residues that encompass MMTV IN β1 and β2 strands (PDB ID: 5D7U) are indicated. The alignment, which was generated using ESPript 3.0 (47), analyzed the following viral sequences: HTLV-1, GenBank access code NC_001436.1; MVV, access code NC_001452.1; RSV, access code J02342.1; PFV, access code U21247.1; HIV-1, access code U26942.1; MMTV, access code NC_001503.1. Numbers mark amino acid positions of indicated IN residues.

Supplementary Figure S11. MMTV WT and IN mutant virus protein content and integration site logos. (**A**) Capsid (CA) content of indicated viruses (delIN = IN deletion control virus); the supernatant of mock-transfected cells was processed in parallel. (**B**) IN content of the indicated viruses. The samples on the leftward blot were immunoprecipitated with affinity-purified rabbit anti-IN antibodies while the samples to the right were treated with normal rabbit control antibodies. The specific IN signal is marked; a more intense non-specific signal, marked by asterisk, was observed in mock samples and samples treated with normal rabbit antibodies. Quantitation of CA and IN signal intensities (average ± s.t.d.) are shown in bar graphs for n=3 independent immunoblotting experiments. Statistical difference versus WT MMTV (***, $p<0.001$) was determined by one-way ANOVA. (**C**) WT and IN mutant base sequence logos surrounding common GTTAAC TSDs (x-axes positions 0 to 5).

Supplementary Figure S12. Distance comparisons of Pro125 analogs among retroviral STCs. Distances between Cα-carbons and N3-nitrogens of an adenosine base in tDNA are displayed. Panels refer to (**A**) PFV (PDB ID: 3OS0) IN Ala188 (tDNA base mutated to adenine *in silico*), (**B**) HIV-1 (PDB ID: 5U1C) IN Ser119 (tDNA base mutated to adenine *in silico*), (**C**) RSV (PDB ID: 5EJK) IN Ser124, (**D**) MMTV IN Pro125 (this study).

Supplementary Table S1. Cryo-EM data collection, refinement and validation statistics.

**Data collection and processing**	**MMTV STC**	**Stacked double-STC**
Microscope	Titan Krios	Titan Krios
Detector	Gatan K2	Gatan K2
Recording mode	Counting	Counting
Magnification	22500	22500
Voltage (kV)	300	300
Total electron exposure (e–/Å^2)	67	67
fluence rate (e–/pix/s)	3.32	3.32
Defocus range (μm)	-1.3 – -3.0	-1.3 – -3.0
Pixel size (Å)	1.31	1.31
Number of frames/movie	100	100
Frame rate (frames/sec)	5	5
Number of micrographs (no.)	1,578	1,578
Symmetry imposed	C2	C1
Total picked particle (no.)	181,238	181,238
Particles used for final map (no.)	50,196	86,379
Map resolution global (FSC 0.143, Å)	3.5	3.8
Map resolution in CIC (FSC 0.143, Å)	3.1 – 4.5	3.3 – 5.5
Directional resolution range (FSC	3.4 – 3.6	3.8 – 3.9
SCF value*	0.97	0.99
**Refinement**		
Refinement package (Real space	Phenix	Phenix
Initial model used	PDB 3JCA and PDB 5CZ2	
Map sharpening B-factor (Å^2)	46	74
Model composition		
Non-hydrogen atoms	18992	31331
Protein (residues)	1984	3147
DNA (nucleotides)	152	298
Map CC	0.80	0.82
FSC map-to-model (global) (0.5)	3.8	4.1
R.m.s deviations		
Bond angles (°)	0.561	0.553
All-atom clashscore	8.87	10.91
Molprobity score	1.57	1.81
Ramachandran plot		
Favored (%)	97.52	96.22
Allowed (%)	2.48	3.78
Outliers	0	0
Rotamer outliers (%)	0.12	0
CaBLAM outliers (%)	2.4	2.2
C-β deviations (%)	0	0
EM-Ringer Score	2.66	1.73

* The SCF value was calculated as described by Baldwin and Lyumkis (1,2) and does not account for false positive orientation assignments.

Supplementary Table S2. Oligonucleotides for MMTV integration site LM-PCR libraries.

Supplementary Table S3. Statistical analyses of integration site distribution in the human genome.

Supplementary References

1. Baldwin,P.R. and Lyumkis,D. (2020) Non-uniformity of projection distributions attenuates resolution in Cryo-EM. *Prog. Biophys. Mol. Biol.*, **150,** 160–183.

2. Baldwin,P.R. and Lyumkis,D. (2021) Tools for visualizing and analyzing Fourier space sampling in Cryo-EM. *Prog. Biophys. Mol. Biol.*, **160,** 53–65.

Supplementary Figure 1

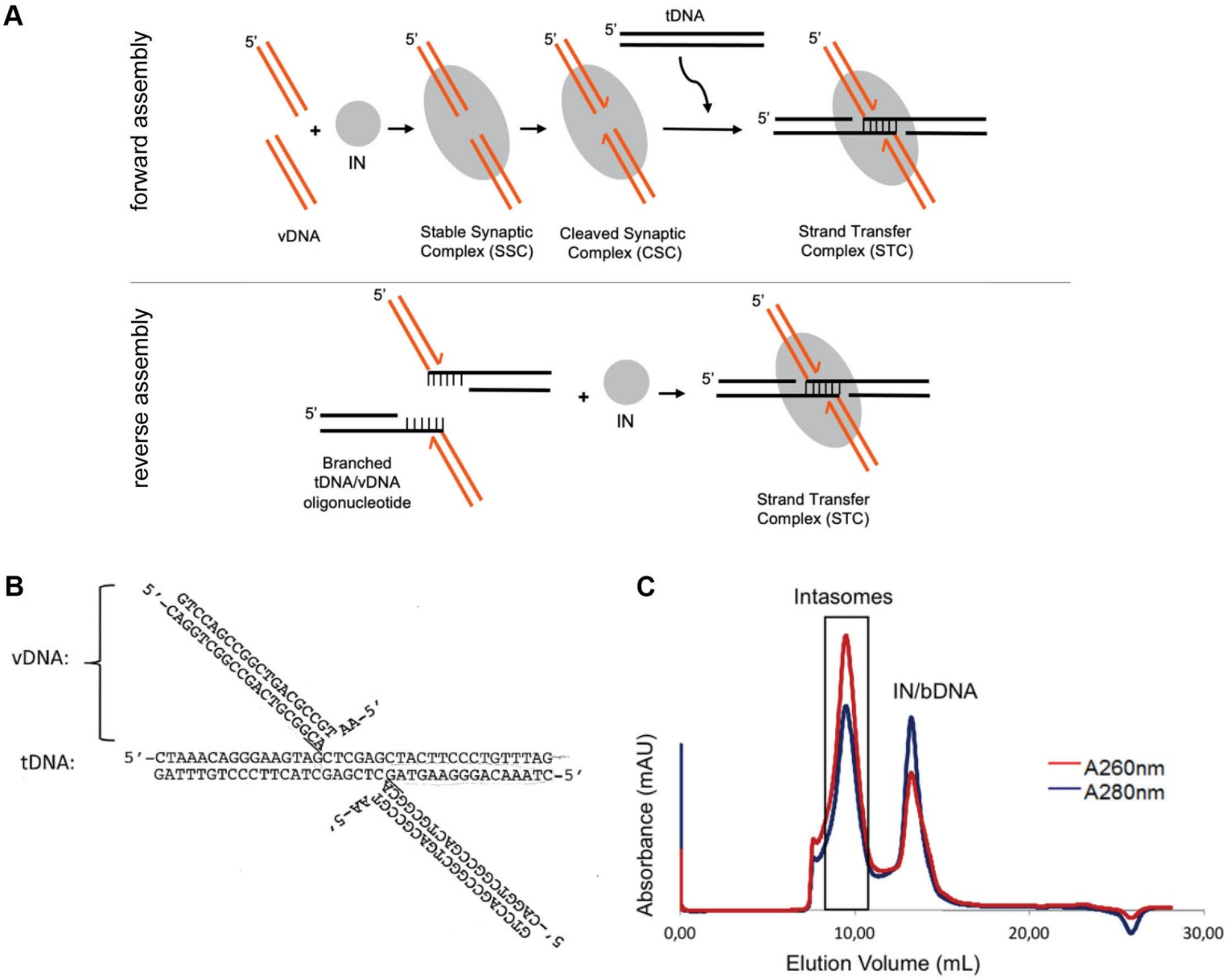

Supplementary Figure 2

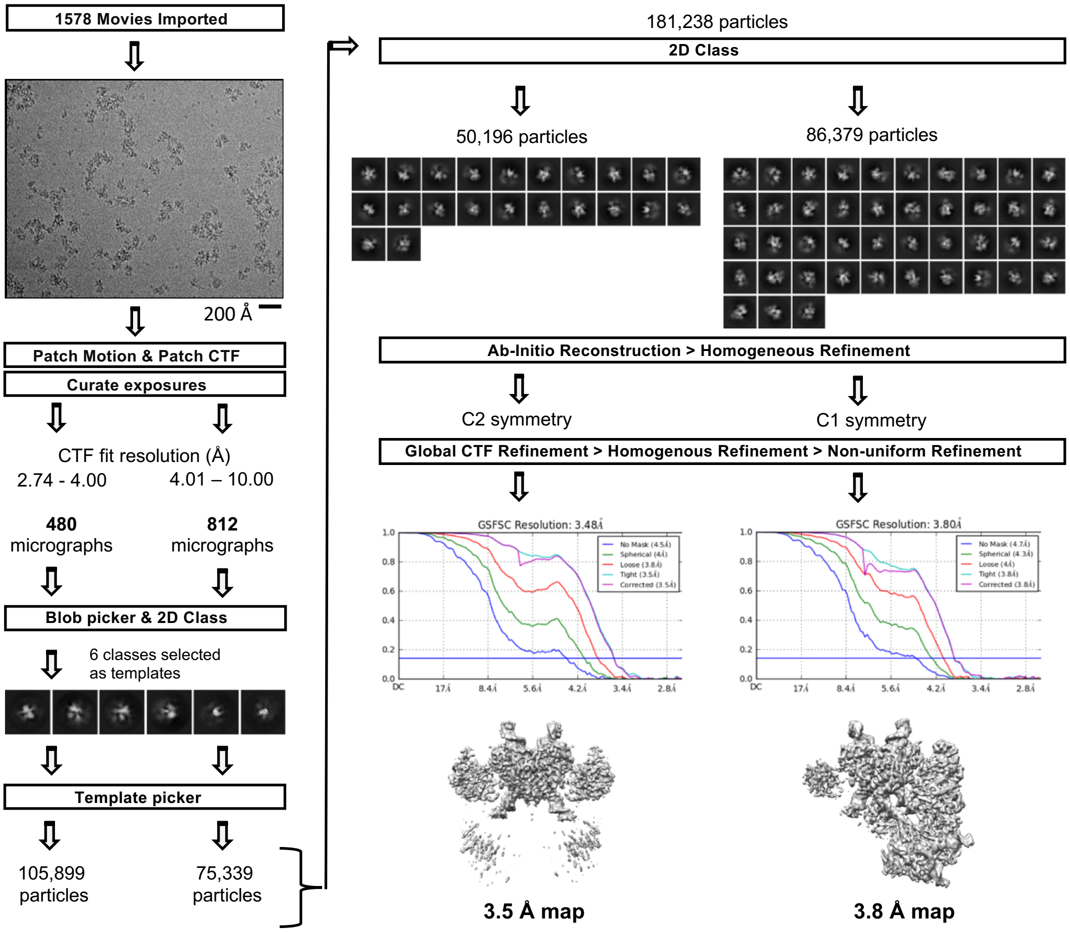

Supplementary Figure 3

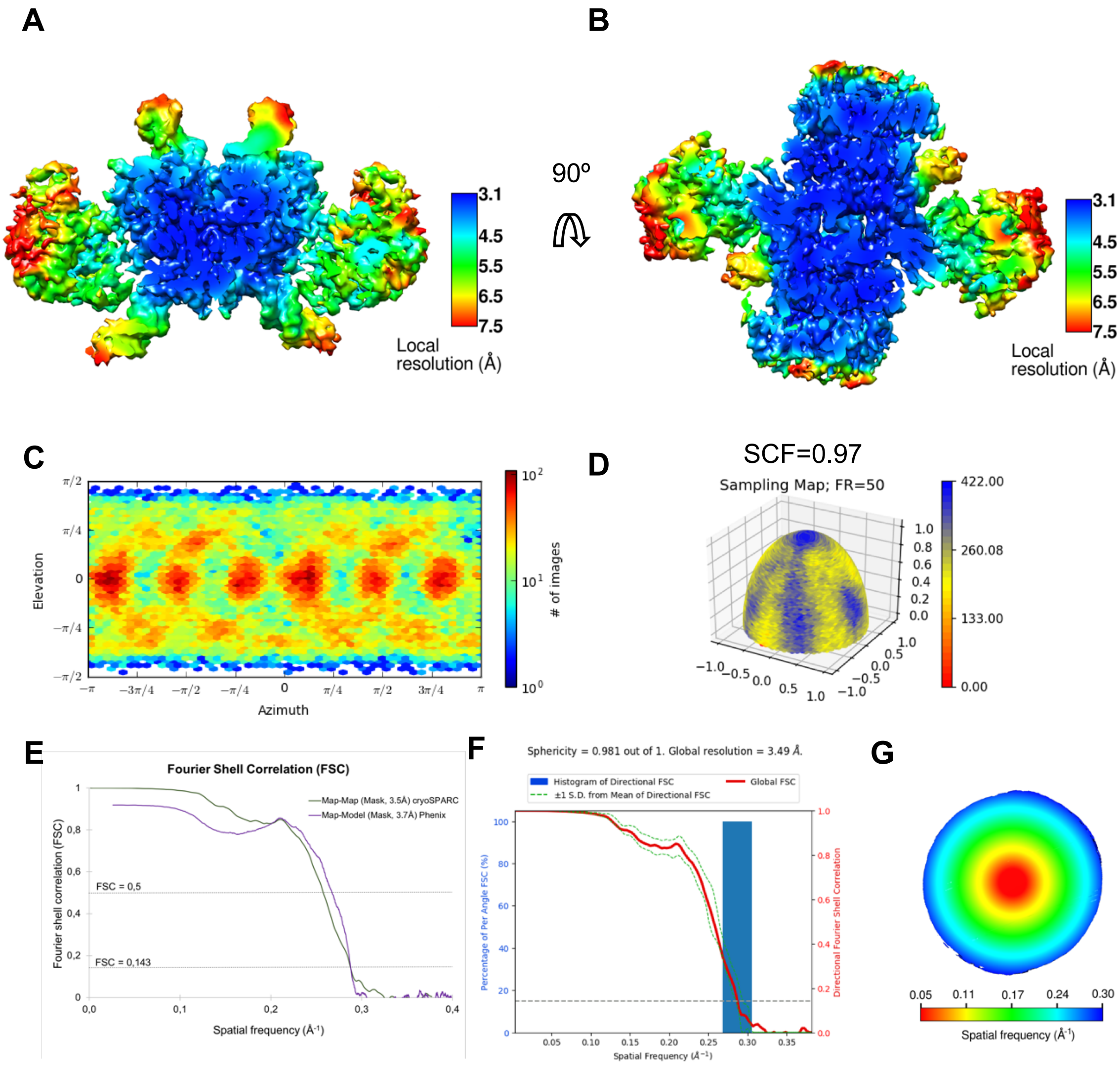

Supplementary Figure 4

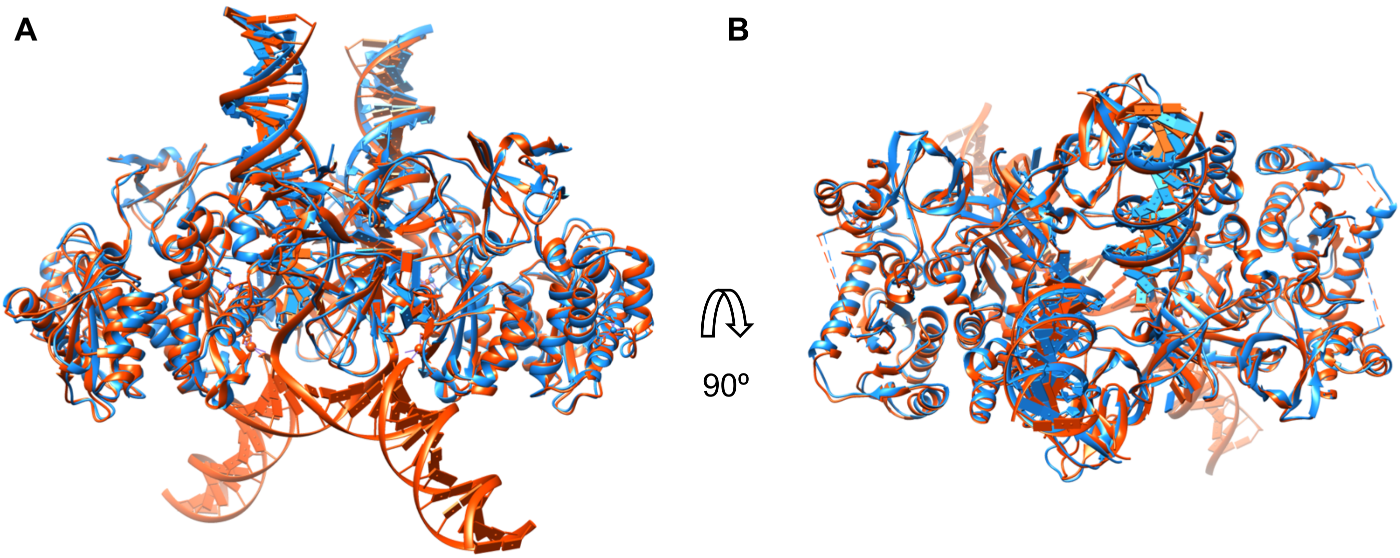

Supplementary Figure 5

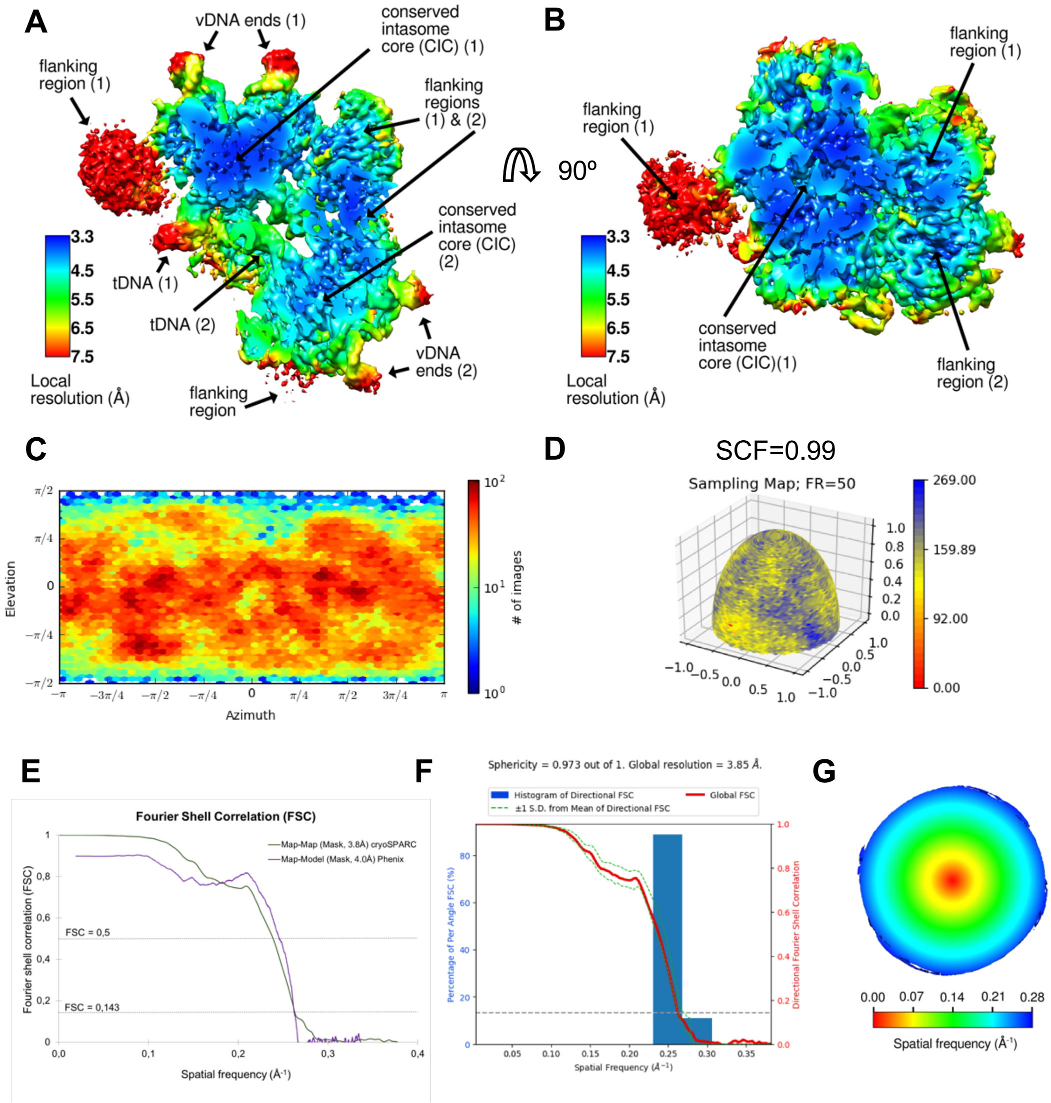

Supplementary Figure 6

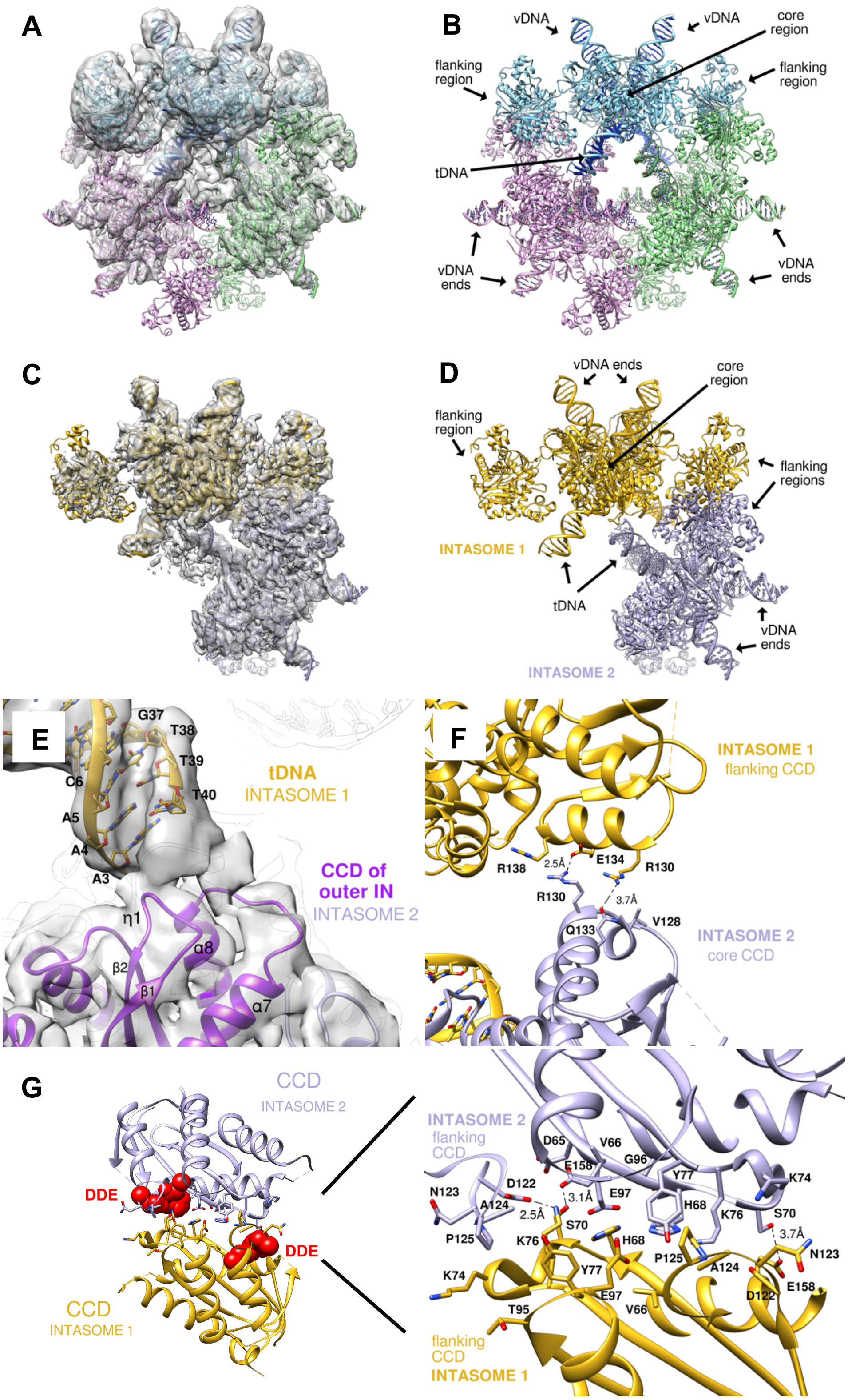

Supplementary Figure 7

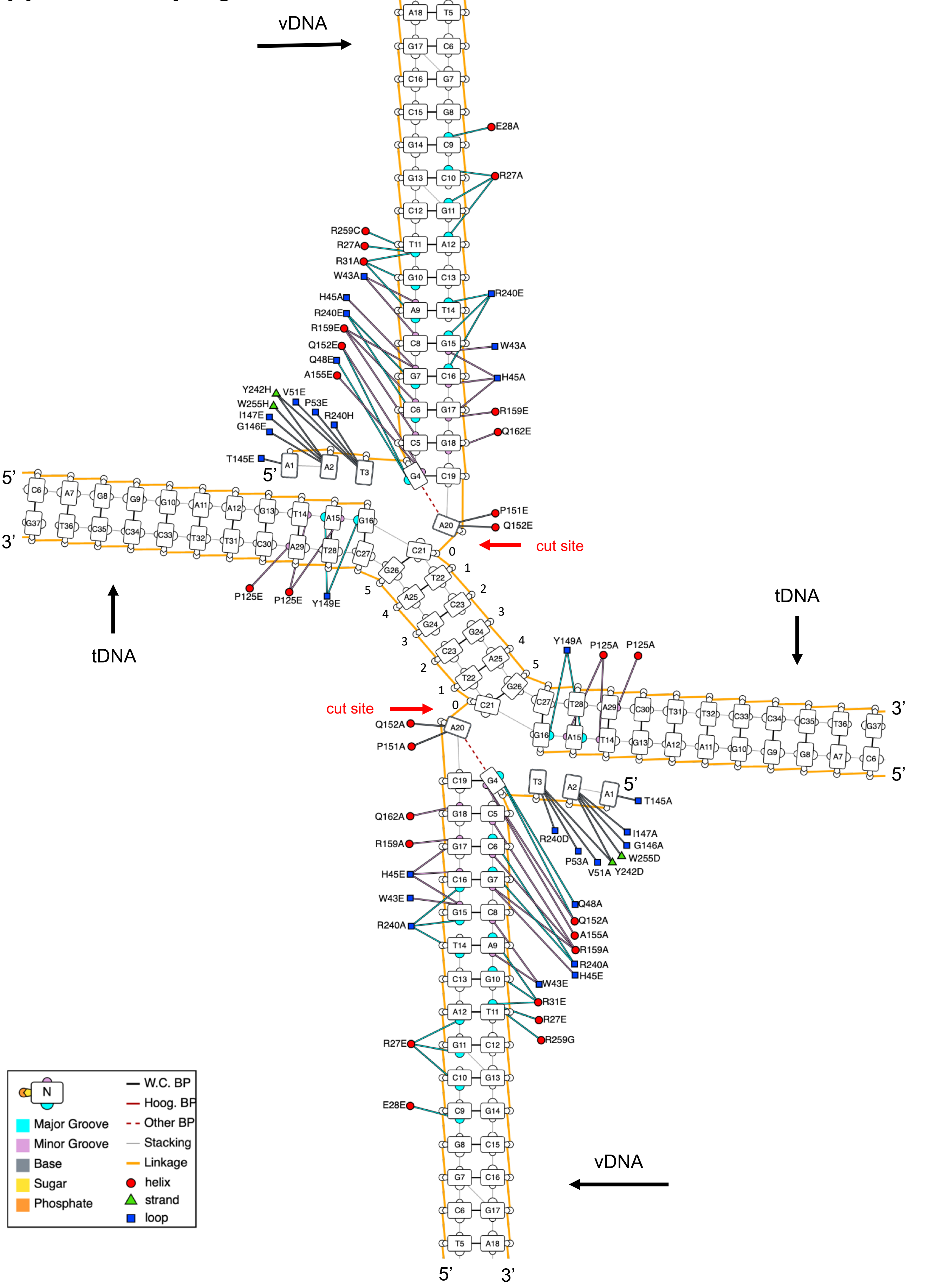

Supplementary Figure 8

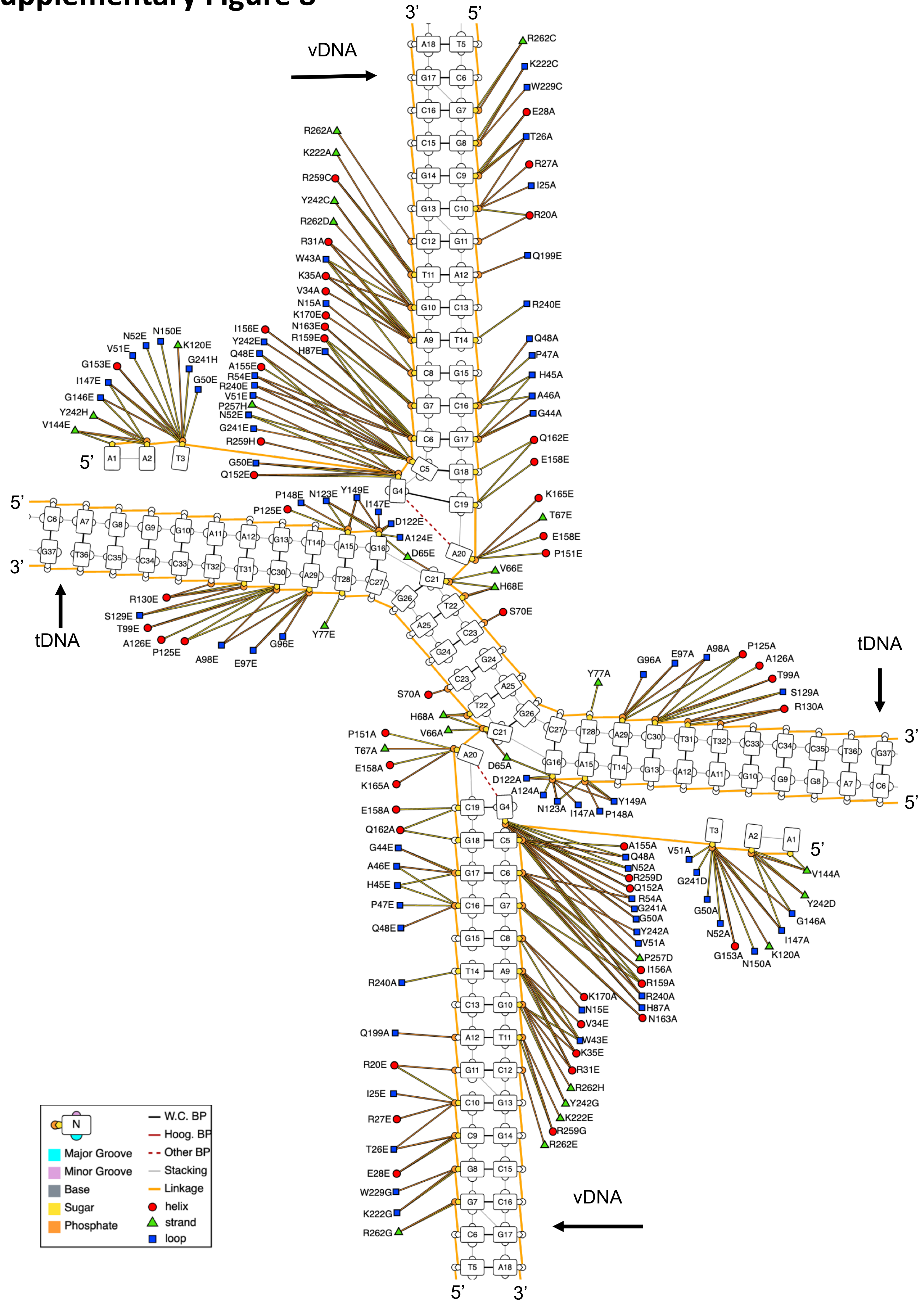

Supplementary Figure 9

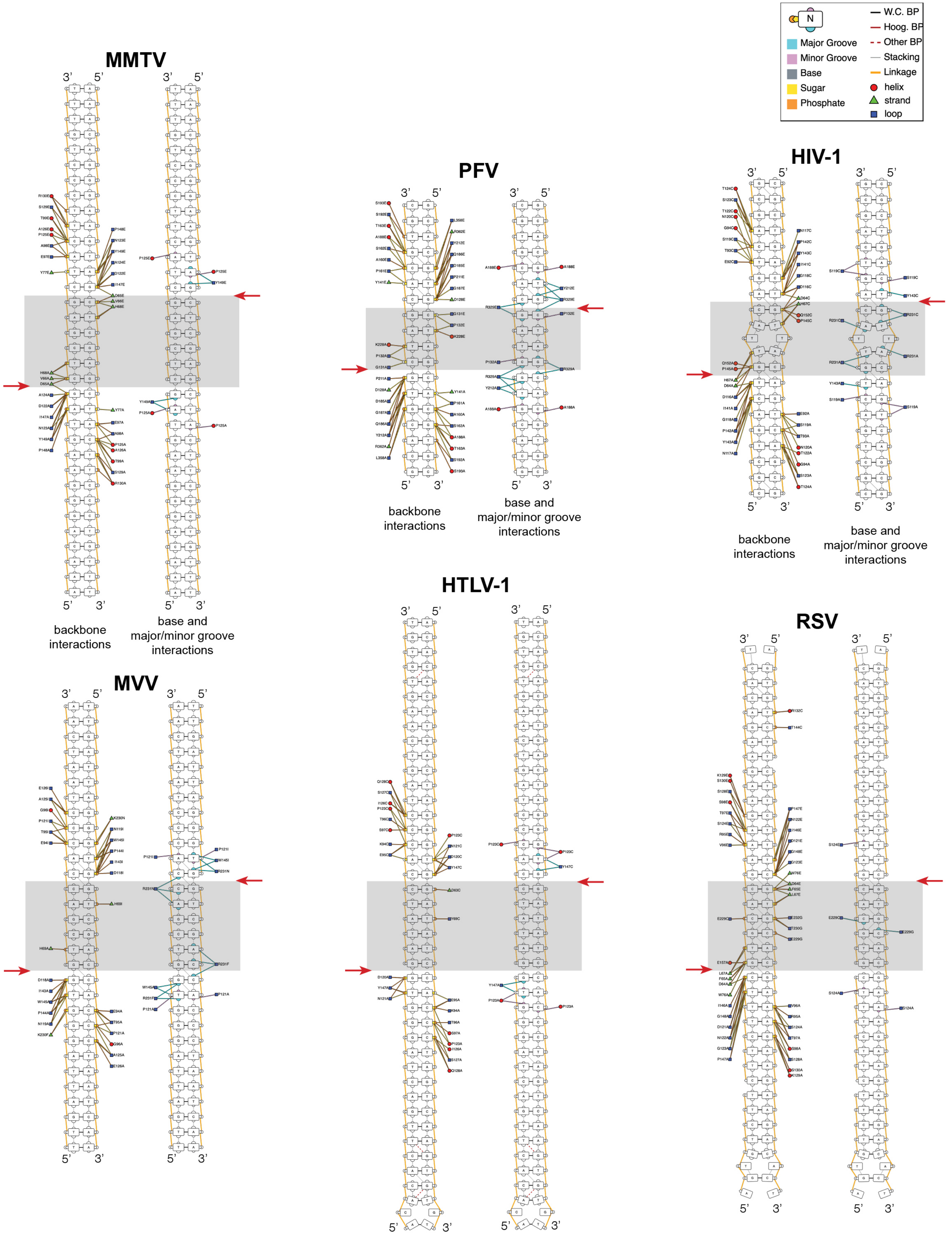

Supplementary Figure 10

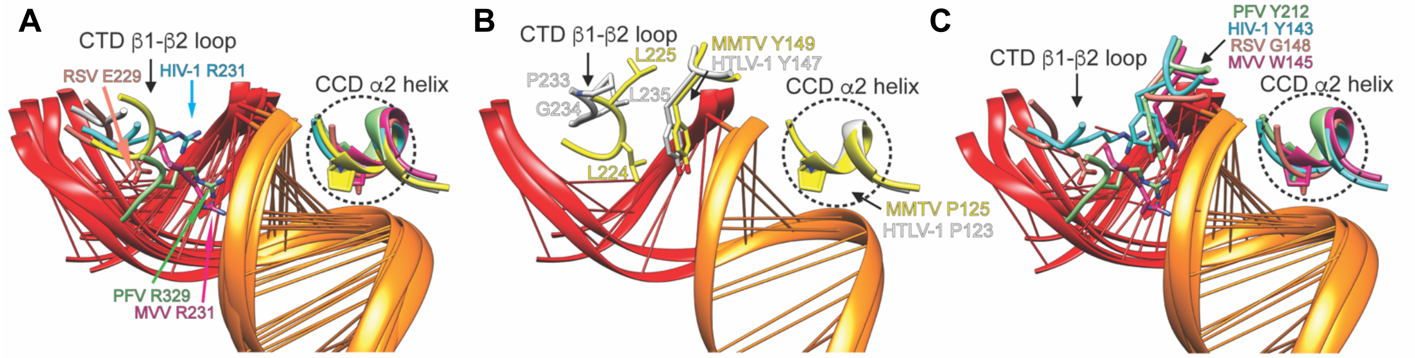

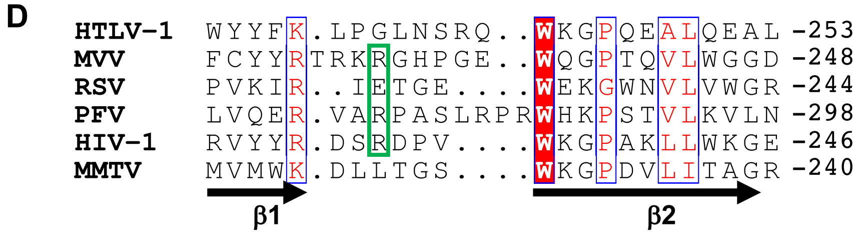

Supplementary Figure 11

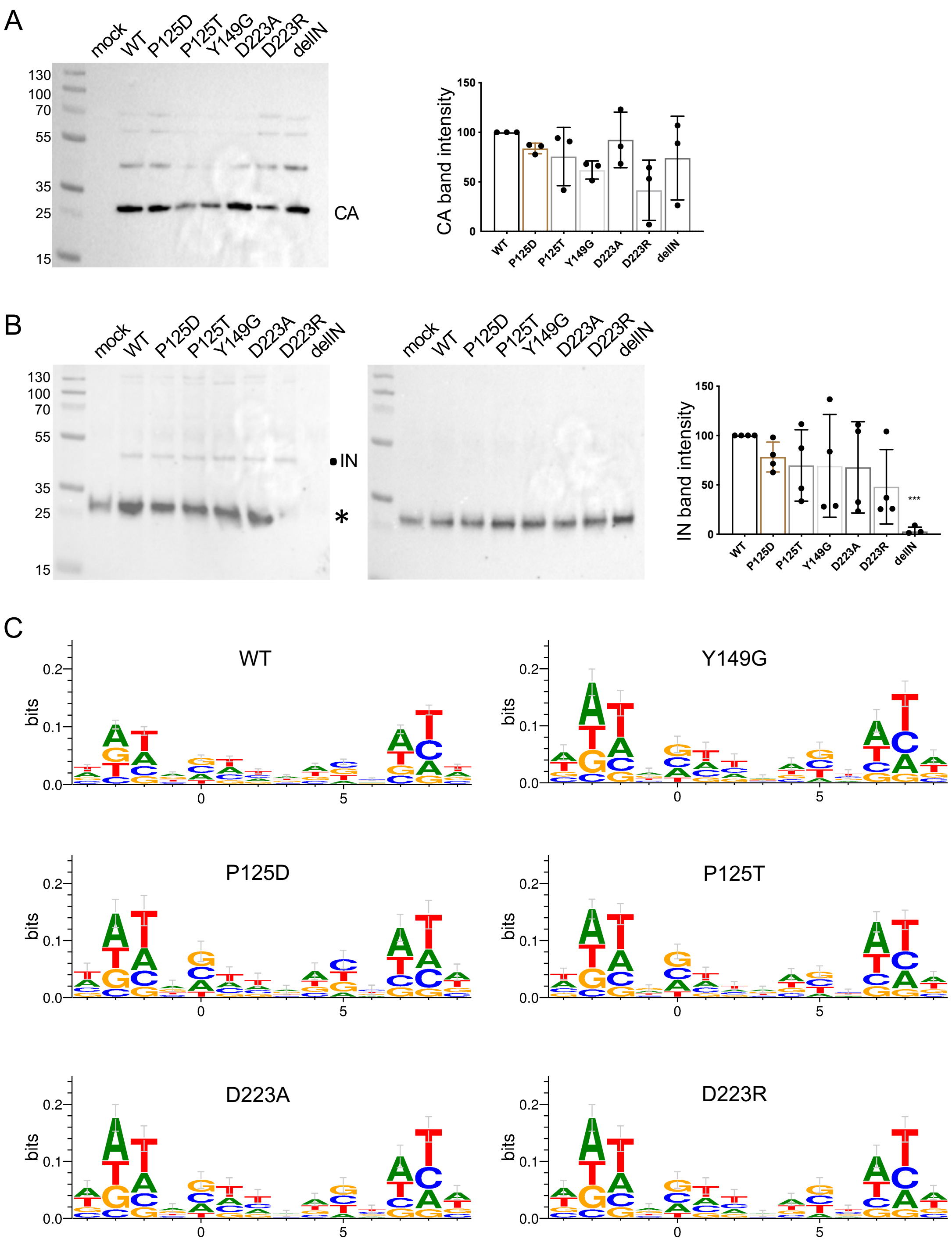

Supplementary Figure 12

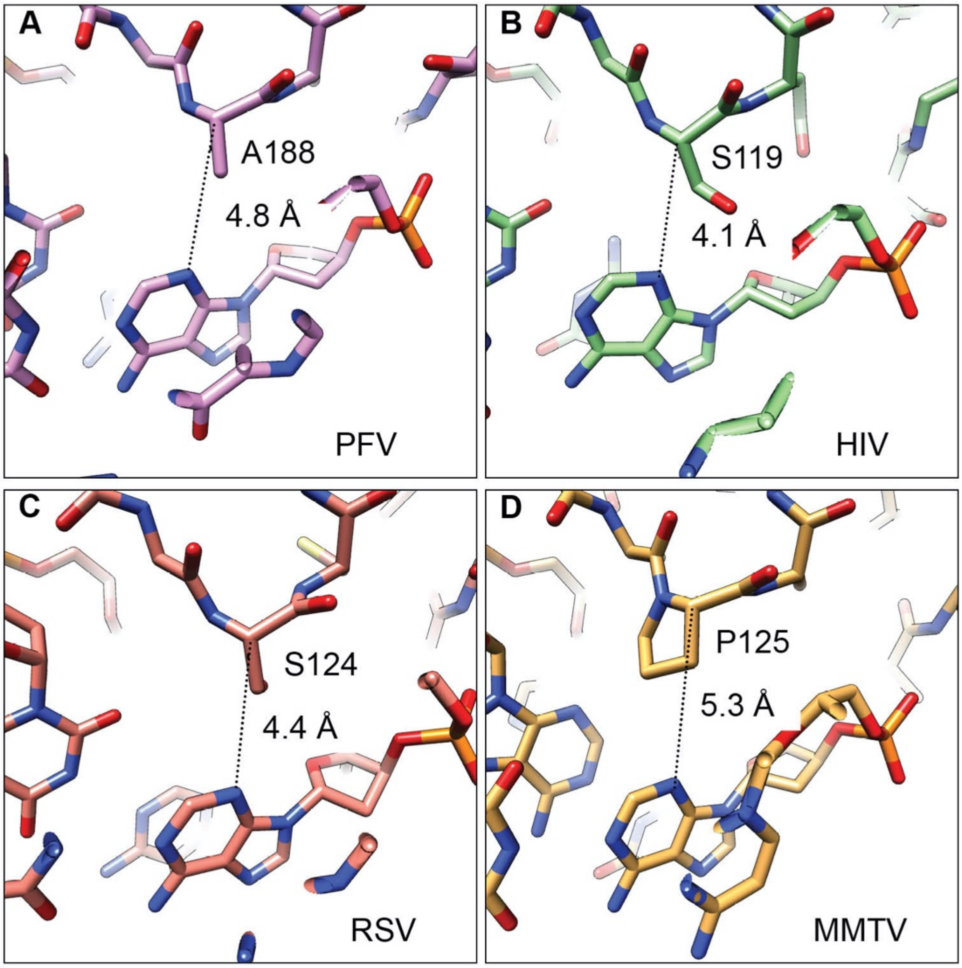

Supplement: gkac644_Supplemental_Files [file gkac644_supplemental_files.zip › gkac644_Supplementary_Data.pdf]
